# Rapid Tagging of Human Proteins with Fluorescent Reporters by Genome Engineering using Double-Stranded DNA Donors

**DOI:** 10.1002/cpmb.102

**Published:** 2019-12

**Authors:** Alexandre Paix, Dominique Rasoloson, Andrew Folkmann, Geraldine Seydoux

**Affiliations:** 1 Department of Molecular Biology and Genetics, Howard Hughes Medical Institute, The Johns Hopkins University School of Medicine, Baltimore, Maryland; 2 Current address: European Molecular Biology Laboratory, Heidelberg, Germany; 3 Corresponding authors: apaix@jhmi.edu; gseydoux@jhmi.edu

**Keywords:** Cas9, CRISPR, double-stranded DNA donors, fluorescent protein, genome engineering, homology-directed repair, tissue culture cells

## Abstract

Tagging proteins with fluorescent reporters such as green fluorescent protein (GFP) is a powerful method to determine protein localization, especially when proteins are tagged in the endogenous context to preserve native genomic regulation. However, insertion of fluorescent reporters into the genomes of mammalian cells has required the construction of plasmids containing selection markers and/or extended sequences homologous to the site of insertion (homology arms). Here we describe a streamlined protocol that eliminates all cloning steps by taking advantage of the high propensity of linear DNAs to engage in homology-directed repair of DNA breaks induced by the Cas9 RNA-guided endonuclease. The protocol uses PCR amplicons, or synthetic gene fragments, with short homology arms (30–40 bp) to insert fluorescent reporters at specific genomic locations. The linear DNAs are introduced into cells with preassembled Cas9-crRNA-tracrRNA complexes using one of two transfection procedures, nucleofection or lipofection. The protocol can be completed under a week, with efficiencies ranging from 0.5% to 20% of transfected cells depending on the locus targeted.

## INTRODUCTION

### A Fast and Cloning-Free Genome Engineering Method

The clustered regularly interspaced short palindromic repeat (CRISPR)/Cas9 technique has emerged as a powerful tool for genome engineering (Doudna & Charpentier, 2014). The Cas9 protein is an RNA-guided endonuclease that can be programmed to target a unique DNA sequence in the genome for cleavage. Such programming requires two small RNAs: a CRISPR RNA (crRNA), comprising a nucleotide sequence complementary to the target DNA, and a trans-activating crRNA (tracrRNA) that serves as a binding scaffold for the Cas9 nuclease and the crRNA. The crRNA and tracrRNA are sometimes combined in a single RNA, called a guide RNA (gRNA; Jinek et al., 2012). The Cas9-crRNA-tracrRNA complex creates a double-strand break that is repaired by the cell’s endogenous homology-directed repair (HDR) system using a synthetic donor DNA containing the desired edit. This method allows deletion, tagging, or mutation of any locus in the genome. One powerful application of genome engineering is the tagging of proteins with fluorescent reporters such as green fluorescent protein (GFP). For this application, the donor DNA consists of a fluorescent reporter–encoding sequence flanked by homology arms matching the sequence around the Cas9-induced cleavage site. Previously published GFP tagging protocols require cloning to create GFP donors with long homology arms or selection schemes to identify homozygous edits (Bollen, Post, Koo, & Snippert, 2018; Danner et al., 2017; Koch et al., 2018; Kwart, Paquet, Teo, & Tessier-Lavigne, 2017), or else require engineered cell lines (Leonetti, Sekine, Kamiyama, Weissman, & Huang, 2016).

**Figure 1:**
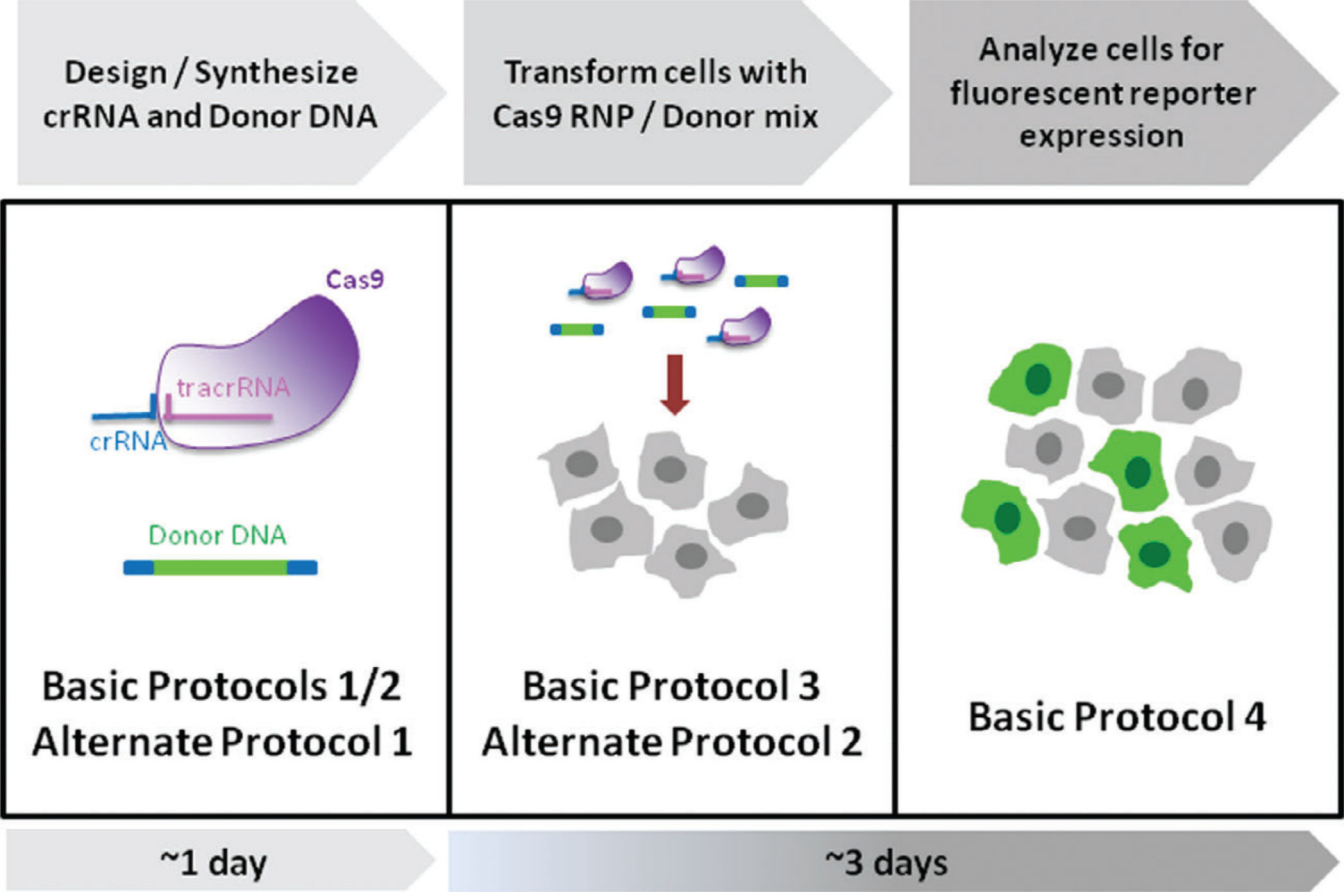
Flowchart of the protocol. Step 1 (left): Design, ordering, and synthesis of required RNA components (the constant tracrRNA component and the variable crRNA component programmed to cut at the target locus), the Cas9 protein and the donor DNA repair template. It takes approximately 1 week between crRNA order and delivery. Step 2 (middle): Generation of the active Cas9 ribonucleoprotein (RNP) complex containing Cas9 protein, crRNA, and tracRNA, followed by the addition of DNA repair template, and cell transformation. Step 3 (right): Identification of targeted genome engineering events by fluorescent reporter expression using cytometry and microscopy.

**Figure 2:**
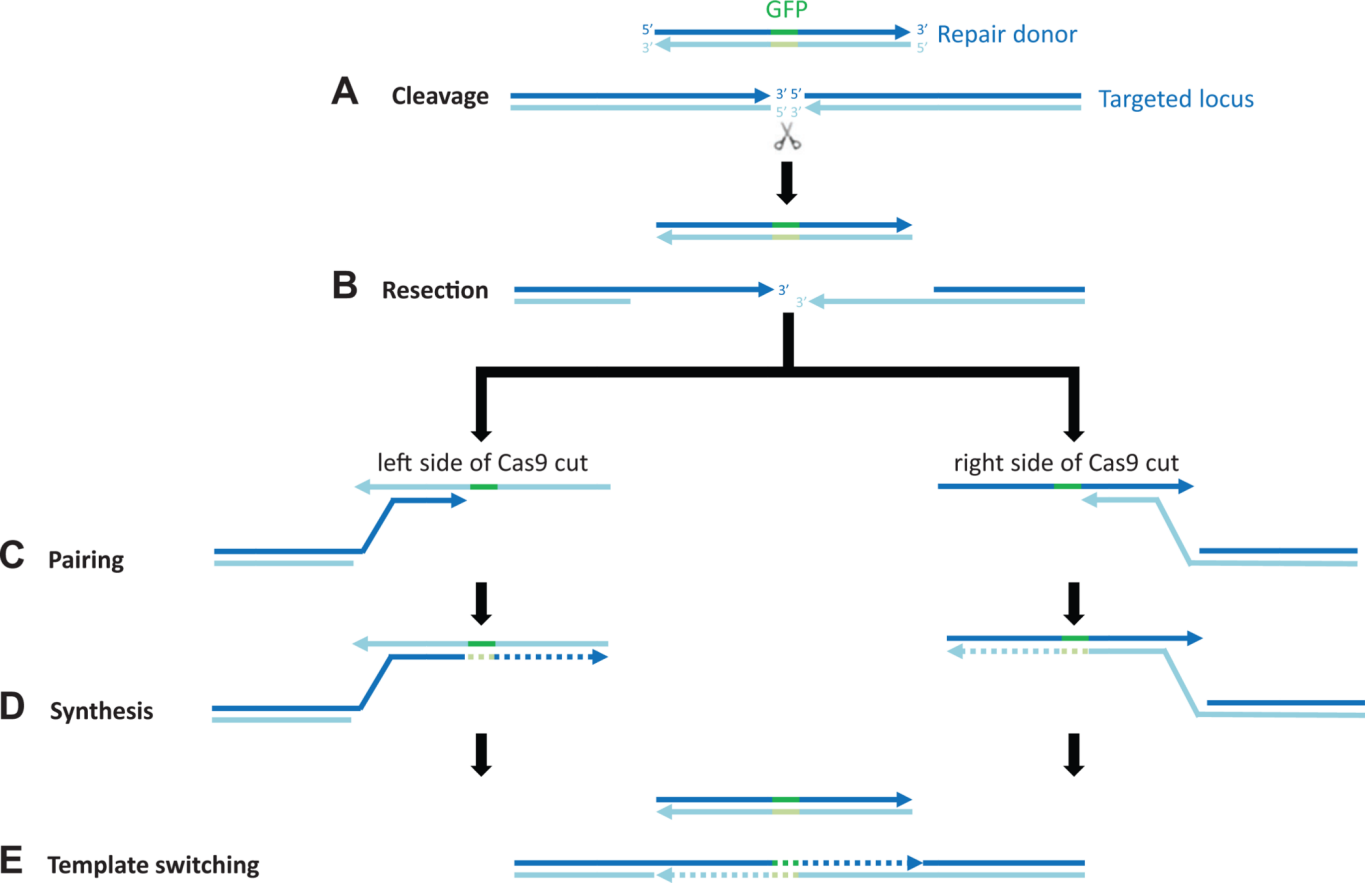
Cas9 cleavage repair using a DNA donor. Schematics show the repair of a DNA break induced by Cas9 (scissor). The DNA of the targeted locus and repair donor are designated by light and dark lines representing the two antiparallel DNA strands. The donor contains a GFP insert (green) and two homology arms (blue). Arrows indicate 3′ ends. (**A**) Cleavage: Cas9 RNP (scissors) creates a double-strand DNA break at the targeted locus. (**B**) Resection: the DNA break is resected by 5′ exonucleases to create 3′ overhangs on each side. (**C**) Pairing: The 3′ overhang strands pair with the repair donor, shown here as two separated DNA strands for clarity. (**D**) Synthesis: The 3′ overhangs are used as primers to begin DNA synthesis (dotted lines) using the repair donor as a template. (**E**) Template switching: The newly synthesized strands dissociate from the template and pair with each other at the locus. Note that the repair donor is not inserted at the locus. This diagram depicts an idealized situation where both overhangs pair with the repair donor. Pairing could also initiate on only one side of the Cas9-induced break to generate only one newly synthesized strand. That strand would then be replicated after template switching back to the locus. See Paix, Folkmann, Goldman, et al. (2017) for more details.

The method described here bypasses the cloning, selection, and engineered cell line requirements by taking advantage of the high propensity of linear DNA fragments with short homology arms to act as templates for the homology-directed repair of Cas9-induced DNA breaks (Figs. 1 and 2; Arbab, Srinivasan, Hashimoto, Geijsen, & Sherwood, 2015; Liang, Potter, Kumar, Ravinder, & Chesnut, 2017; Paix, Folkmann, Goldman, et al., 2017; Paix, Folkmann, Rasoloson, & Seydoux, 2015; Paix, Schmidt, & Seydoux, 2016; Paix et al., 2014). The high editing frequency allows genomic modifications to be identified directly without selection by screening for cells with fluorescent reporter expression. Procedures requiring cloning are also eliminated by using recombinant Cas9 protein and chemically synthesized crRNAs and tracrRNAs that are assembled with Cas9 protein in vitro before being introduced into cells (Lin, Staahl, Alla, & Doudna, 2014; Paix, Folkmann, Goldman, et al., 2017).

**Figure 3:**
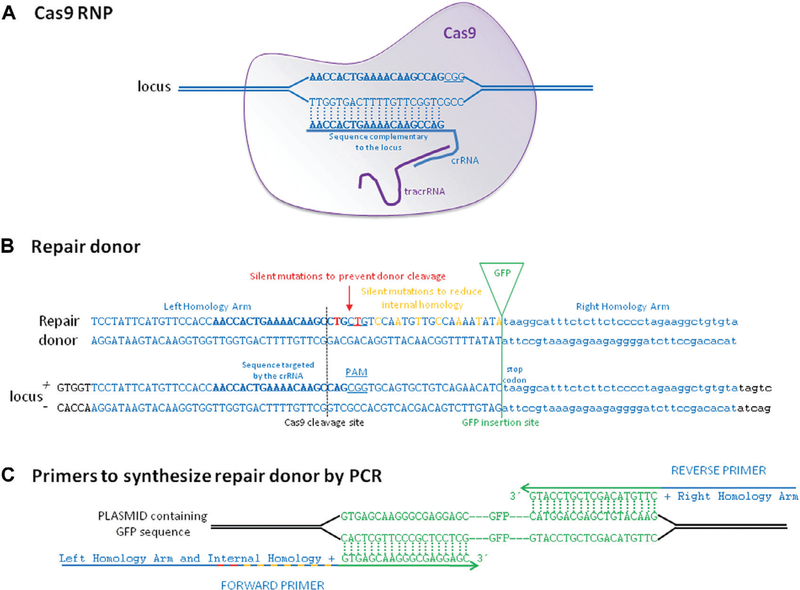
Reagents for Cas9 RNP genome engineering. (**A**) Cas9 RNP: simplified schematic of CRISPR/Cas9 components (Cas9 protein, tracrRNA, and crRNA) required to target a specific genomic locus. The crRNA contains a nonvariable sequence (blue line) and a sequence complementary to the locus (bold) and immediately upstream of a protospacer-adjacent motif (PAM) in the locus (CGG with overbar in the example shown). The tracrRNA (purple) binds Cas9 and the nonvariable sequence of the crRNA to form a functional Cas9 ribonucleoprotein complex (RNP) that will cleave the targeted locus. See Basic Protocol 1 for assembly of the Cas9 RNP. (**B**) Repair donor: in this example, the donor is designed to insert the GFP coding sequence just upstream of the STOP codon (“taa”). The sequence targeted by the crRNA (spacer) is in bold and the PAM is underlined. In this example, the Cas9 cleavage site is 26 nt away from the desired GFP insertion site, which is at the upper limit for successful editing. Mutations introduced to prevent donor DNA cleavage by Cas9 or to reduce internal homology are indicated. Note that these mutations are not required in donors where the site for GFP insertion resides in the PAM or the sequence targeted by the crRNA. (**C**) Primers to generate the repair donor using PCR: the GFP coding sequence (green) in a plasmid template (black, Figure 7 and Table S2) is amplified by PCR using forward and reverse primers. The primers are complementary at their 3′ ends with the GFP sequence (green) and contain flanking sequences homologous to the targeted locus as shown in (B). See Basic Protocol 2 for details. Note that the repair donor can also be obtained commercially as a gBlock (Alternate Protocol 1).

The method includes three main steps (Fig. 1):

1. Design of reagents (Fig. 3) and synthesis and ordering of required RNA components (crRNA and tracrRNA) and DNA repair templates (Basic Protocols 1 and 2 and Alternate Protocol 1).
2. Introduction of the Cas9 ribonucleoprotein (RNP) complex (Cas9 protein, crRNA, and tracrRNA) and DNA repair template into cells by nucleofection or lipofection (Basic Protocol 3 and Alternate Protocol 2) into cells.
3. Screening and sorting of fluorescent cells by flow cytometry (Basic Protocol 4).

The entire method can be completed in a week. Supporting protocols for Cas9 protein purification (Support Protocol 1) and tissue culture maintenance (Support Protocol 2) are also provided.

### Application of the Method

The main application of this protocol is to tag proteins with a fluorescent reporter such as GFP to analyze protein localization and dynamics in cells (Chudakov, Matz, Lukyanov, & Lukyanov, 2010; Cranfill et al., 2016). The method outlined here can be used to generate multiple fusions in parallel (Tables 1 and 2). The protocol can also be used to estimate the targeting efficiency of a particular crRNA, because the percentage of cells expressing the fluorescent reporter is a function of targeting efficiency at the locus. Several guide RNAs can be easily tested in parallel to identify the most efficient crRNA for subsequent targeting and integration of other tags. Prior knowledge of guide efficiency is particularly useful when choosing guides to create edits that cannot be identified by fluorescence, including insertion of short immunogenic tags and point mutations. Such edits need to be screened by PCR or sequencing using clonal populations derived from the edited population (Koch et al., 2018; Kwart et al., 2017; Paix, Folkmann, Goldman, et al., 2017; Yang, Yang, Byrne, Pan, & Church, 2014).

### Limitations of the Method

**Figure 4:**
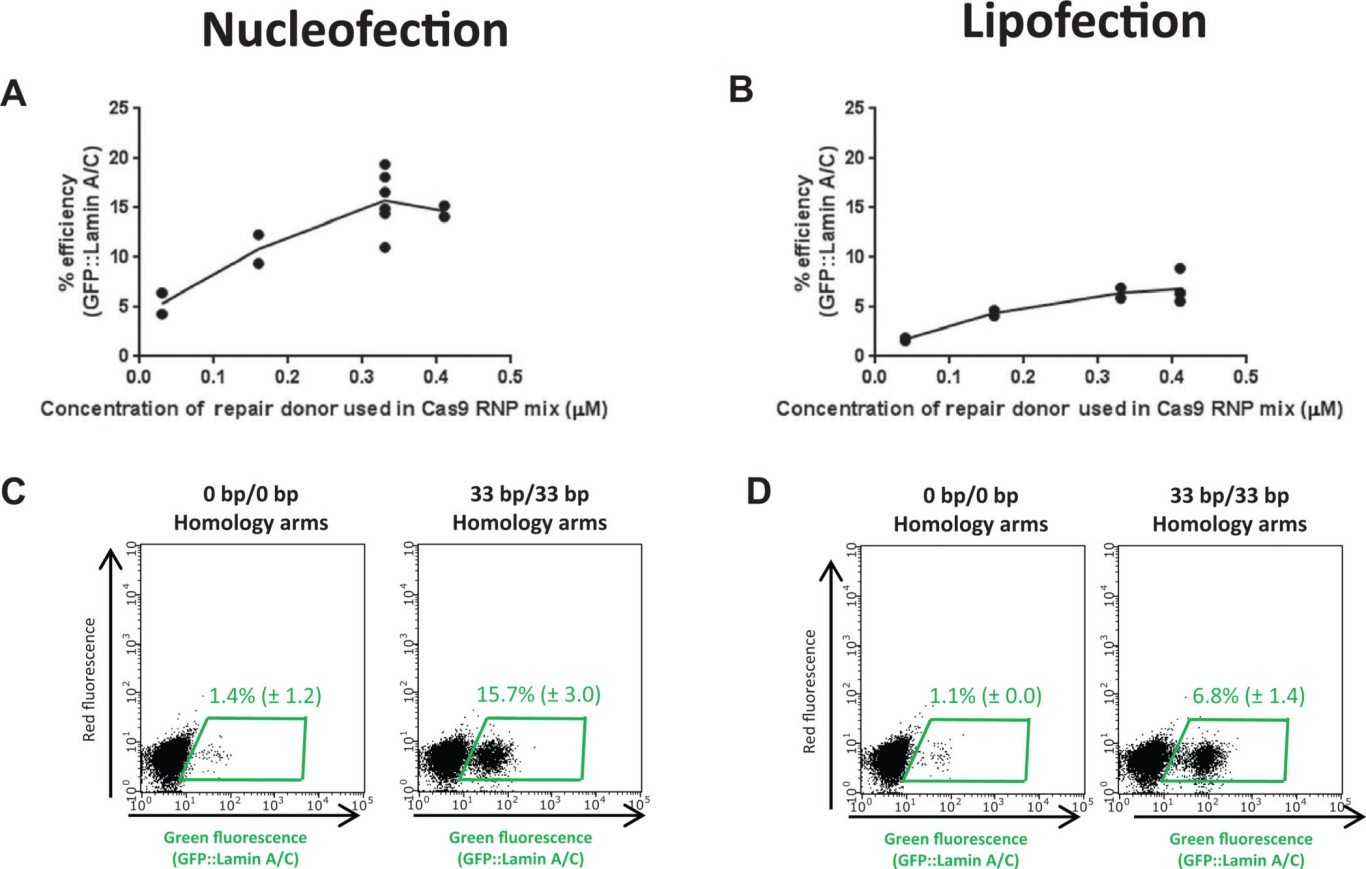
Effect of donor DNA concentration and homology arms on editing efficiency. (**A**) and (**B**) Graphs comparing the efficiency of GFP+ edits in the *Lamin A/C* locus using PCR donors introduced by nucleofection (**A**) or lipofection (**B**) in HEK293T cells. Note that editing efficiency increases with increasing donor concentration in the transformation mix, reaching a plateau at ~0.33 μM. (**C**) and (**D**) Plots showing the number of GFP+ targeted cells obtained by nucleofection (**C**) or lipofection (**D**), as determined by cytometry. Targeting efficiencies were determined using DNA repair donors with no homology arms (0 bp/0 bp) or 33-bp homology arms (33 bp/33 bp). The repair donors with no homology arms were used to estimate rates of false positives for homology-directed repair.

**Figure 5:**
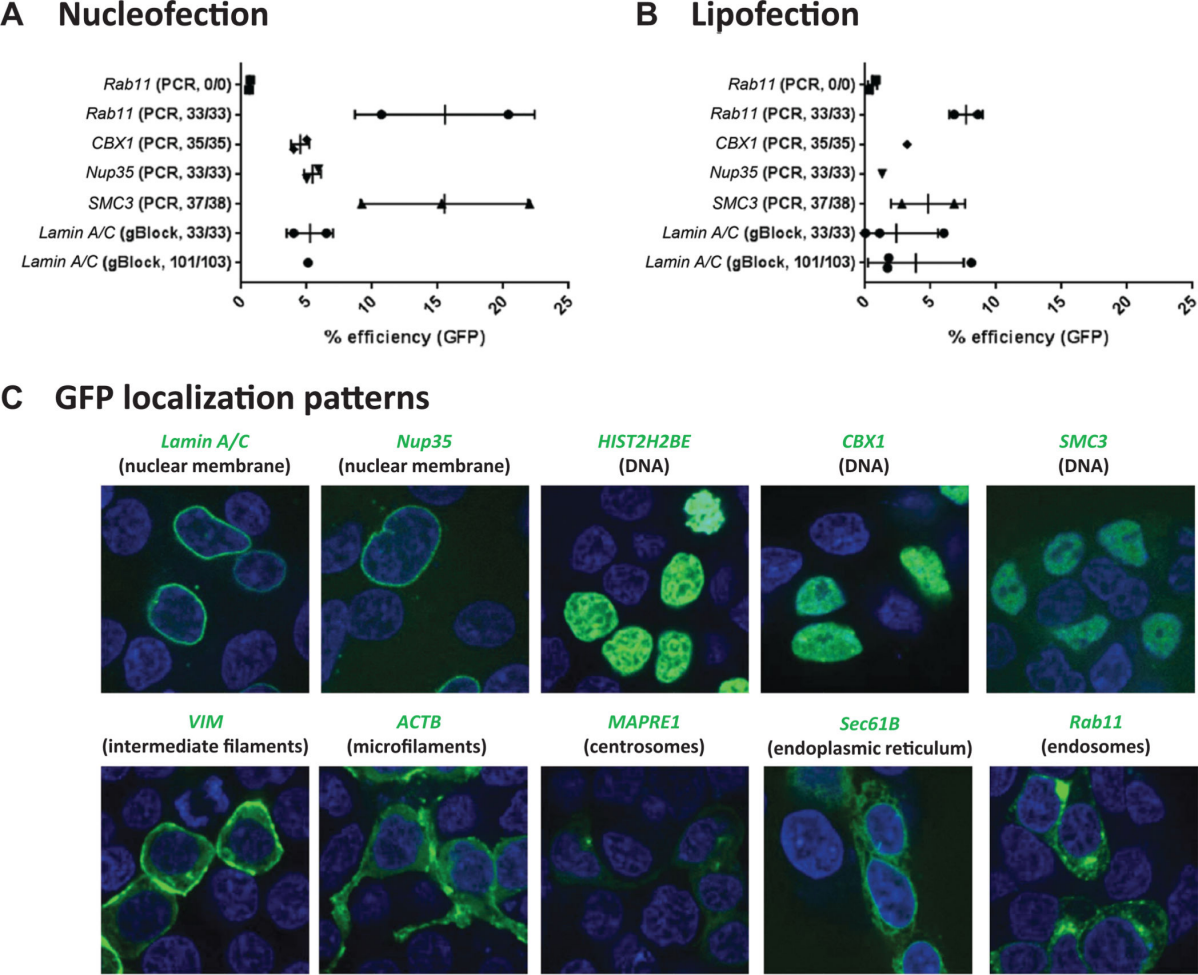
Tagging efficiencies at different loci. (**A**) and (**B**) Graphs showing the percentage of GFP+ HEK293T cells obtained using the indicated donor DNAs. The type of donor DNA (PCR or gBlock) and the length of the homology arms are indicated in parenthesis. Efficiencies are higher when using nucleofection (**A**) than when using lipofection (**B**). (**C**) Example of GFP localization patterns in HEK293 cells. GFP signal is in green and nuclear DNA staining in blue. Localizations are indicated below each targeted gene.

**Figure 6:**
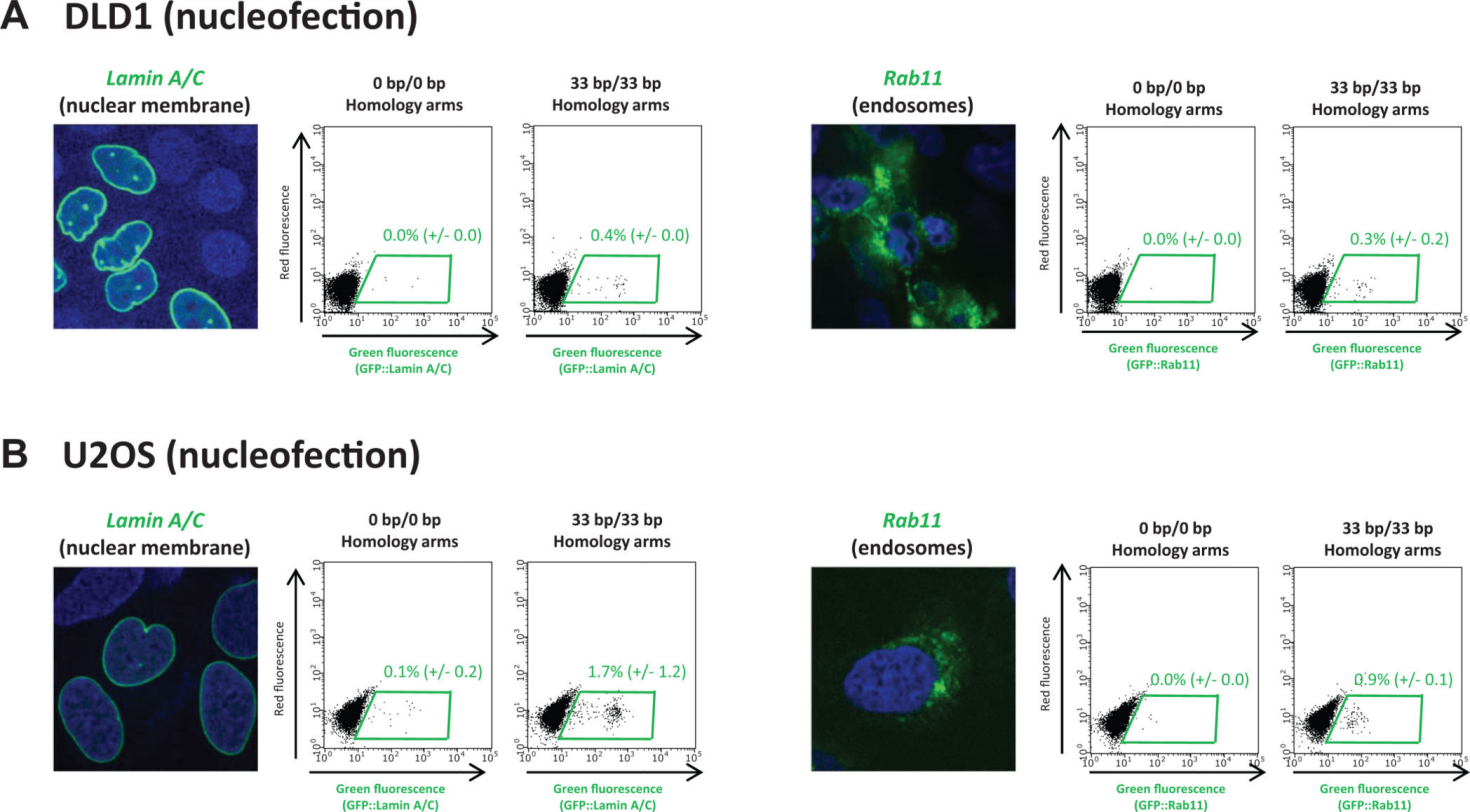
Gene tagging in DLD1 and U2OS cells. (**A**) DLD1 cells edited to insert GFP in the *Lamin A/C* and *Rab11* loci using nucleofection and PCR donors. Plots show the number of GFP+ cells, as determined by cytometry, comparing PCR donors with no homology arms (0 bp/0 bp) and with 33-bp homology arms (33 bp/33 bp). The repair donors with no homology arms were used to estimate rates of false positives for homology-directed repair. (**B**) Same as in (**A**) but using U2OS cells.

**Figure 7:**
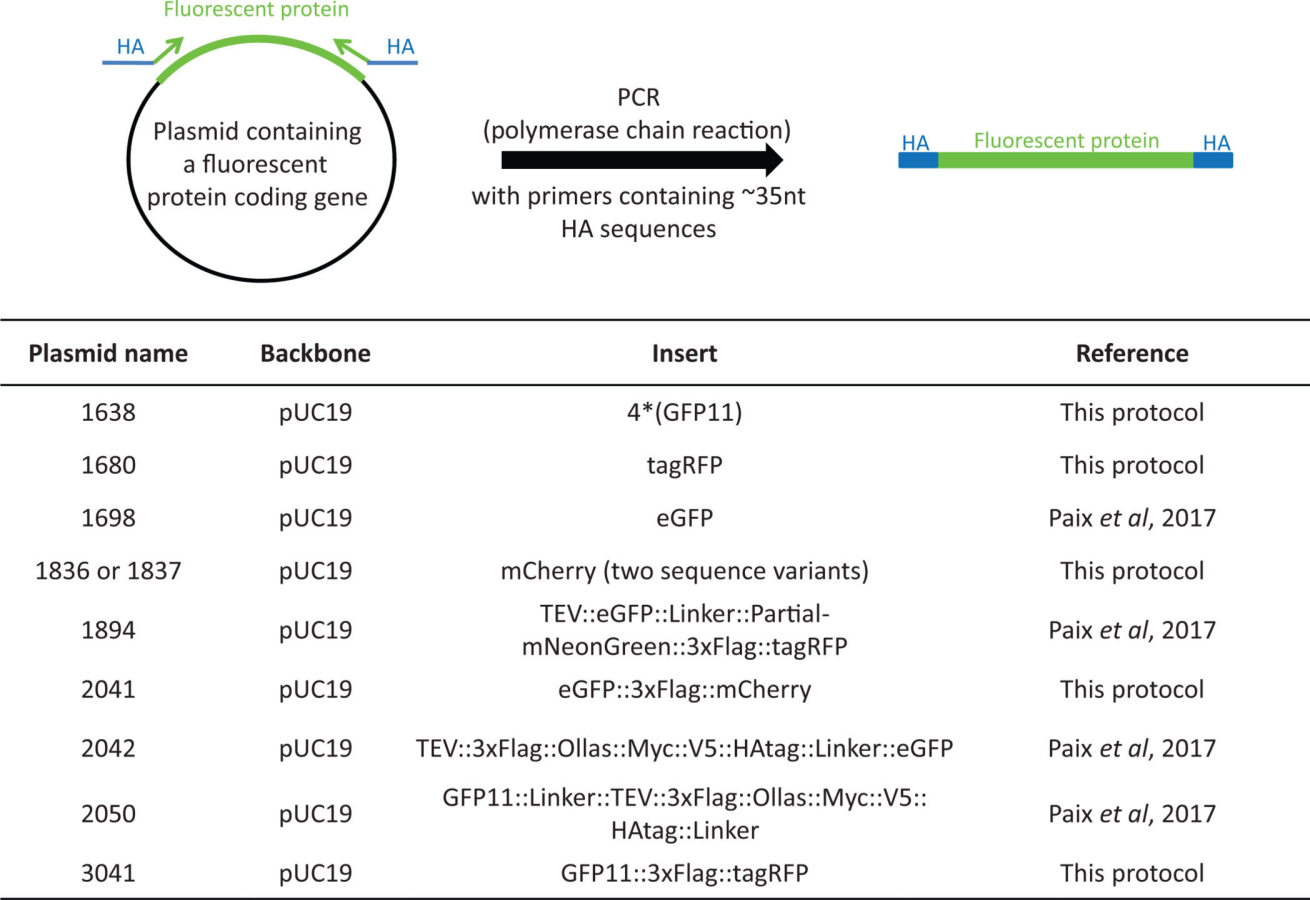
List of plasmids to create donor DNAs by PCR. Repair donors can be amplified using PCR primers annealing with the beginning and the end of the fluorescent reporter (green) and containing short flanking sequences (~35 nt) homologous to the gene to edit (homology arms [HA], blue). Plasmids containing various fluorescent reporters and epitope tags are used as PCR template for amplification (Supplementary Information File S1).

The protocol relies on an efficient type of homology-dependent repair, synthesis-dependent strand annealing (SDSA), in which donor DNAs are used as templates for repair DNA synthesis (Fig. 2; Jasin & Haber, 2016; Mehta, Beach, & Haber, 2017; Paques & Haber, 1999). This type of repair makes for an efficient and simple protocol, but some limitations need to be kept in mind:

- SDSA repair is most efficient when the Cas9 cleavage site is within 10 bases of the desired integration site (Paix, Folkmann, Goldman, et al., 2017; Paquet et al., 2016).
- Editing efficiency varies with different cell types, guide RNAs, and transformation methods (details are provided in Figs. 4, 5, 6, and 7, Tables 1 and 2, and Supporting Information,Tables S1–S3). Nucleofection (Basic Protocol 3) is more efficient than lipofection (Alternate Protocol 2) for most cell lines. Some users, however, may prefer lipofection for its ease of use and workable efficiency, especially in HEK293T cells.
- Occasionally, repair using linear templates leads to small insertions or deletions, especially at junctions between the fluorescent reporter and the genomic region around the Cas9 cleavage site (Paix et al., 2017). This is less of a concern when working with polyclonal cell populations, which contain multiple independent edits. When working with clonal populations, however, we recommend sequencing the edited locus (both alleles) to ensure that there are no undesired mutations.
- The method presented here typically results in the tagging of only one allele. Heterozygous tagging is sufficient to analyze protein localization and dynamics, but not to quantify protein levels or to verify the functionality of the tagged protein. For experiments that require homozygous edits, other approaches can be used (Koch et al., 2018; Paquet et al., 2016).

## BASIC PROTOCOL 1

### crRNA DESIGN

Cas9 is an RNA-guided endonuclease that requires a crRNA for targeting to a specific genomic site (Figs. 1–3 and Supporting Information, Table S3; Jinek et al., 2012). The crRNA consists of a sequence of 20 nt homologous to the targeted genomic region (spacer sequence; Fig. 3) and a nonvariable sequence (repeat sequence). The spacer sequence is designed in function of the targeted genomic region and must be upstream of a so-called protospacer-adjacent motif (PAM) in the targeted genomic region. The PAM for the Cas9 protein from *Streptococcus pyogenes* (spyCas9) is the trinucleotide NGG (Fig. 3). The nonvariable repeat sequence of the crRNA is necessary to bind to a second RNA called the tracrRNA. The tracrRNA is a nonvariable RNA molecule that contains a sequence complementary to the crRNA repeat sequence and a sequence required to bind Cas9 protein. The same tracrRNA is used for all experiments; only the crRNA needs to be custom designed. Because single gRNAs (combined crRNA and tracrRNA in one molecule; Jinek et al., 2012) are longer than crRNAs and thus more expensive to synthesize, they are not recommended for this protocol.

#### Materials

Milli-Q-filtered H_2_O, sterile (stored at room temperature)

Ultrapure 1 M Tris Cl buffer, pH 7.5 (e.g., Thermo Fisher Scientific, cat. no. 15567027) and 5 mM working solution (diluted in sterile Milli-Q-filtered H_2_O and stored at room temperature)

Custom-synthesized crRNA (e.g., Integrated DNA Technologies [IDT] Alt-R CRISPR-Cas9 crRNA)

tracrRNA (e.g., IDT CRISPR-Cas9 tracrRNA)

1. Identify the best site for GFP insertion for your particular locus.AC-terminal fusion is often considered least likely to interfere with possible localization sequences that are often located at the N terminus. N-terminal and internal insertion sites can also be considered as long as functional domains are not disturbed.
  We recommend identifying protein domains using online resources such as SMART (http://smart.embl-heidelberg.de/) in order to choose an insertion site that will not disturb an essential domain (Letunic & Bork, 2018).
2. Design the crRNA following these guidelines:
  • Choose a crRNA that will cause Cas9 to cleave the genomic DNA as close as possible to the desired GFP insertion site. Cas9 will generate a blunt cut 3 bp upstream of the PAM sequence. For best results, we recommend staying within a window of ±10 bp from the edit (Paix, Folkmann, Goldman, et al., 2017).
  • When possible, choose a crRNA containing a guanine nucleotide immediately before the PAM. Avoid having a cytosine at that position (Doench et al., 2014).
  • A GC content of 50%−70% GC in the crRNA spacer sequence also improves cutting efficiency (Gagnon et al., 2014).
  • Make sure that the crRNA targets a unique sequence (20 bp) and has few, if any, predicted off-target potential editing sites. Generally, two or more mismatches within the 10 bp upstream of the PAM sequence are sufficient to avoid targeting by Cas9. We recommend using online resources to identify potential off-target sites for crRNAs (https://portals.broadinstitute.org/gpp/public/analysis-tools/sgrna-design; Doench et al., 2016).
3. Order the crRNA: Several commercial sources are available, including IDT Alt-R CRISPR-Cas9 crRNA. Two nanomoles is sufficient for 60 experiments using lipofection or six experiments using nucleofection. If more experiments are planned, order 10 nmol. Use the provider’s website to enter the desired sequence (with IDT, only the 20-nt spacer sequence needs to be entered; do not include the PAM or repeat sequence). Briefly spin the crRNA in a bench microcentrifuge at room temperature and add 30.8 μl 5 mM Tris·Cl, pH 7.5, to the 2 nmol of crRNA provided (final concentration: 65 μM). Mix well by pipetting and store at −80°C.
4. Order the tracrRNA: Use the same provider as for your crRNA to make sure that the crRNA and tracrRNA will be able to pair. Twenty nanomoles is sufficient for 600 experiments using lipofection or 60 experiments using nucleofection. Briefly spin the tracrRNA in a bench microcentrifuge at room temperature and add 308 μl 5 mM Tris·Cl, pH 7.5, to the 20 nmol of tracrRNA provided (final concentration: 65 μM). Mix well by pipetting and store at −80°C.

## BASIC PROTOCOL 2

### PCR DONOR DESIGN AND SYNTHESIS

Insertion of a fluorescent reporter is achieved using a linear donor DNA that will function as a template to repair the Cas9-induced break through homology-directed repair (Paix, Folkmann, Goldman, et al., 2017). Linear donors are synthesized in the lab using the polymerase chain reaction (PCR) or can be purchased as gene fragments (e.g., IDT gBlocks; Alternate Protocol 1).

The donor template consists of the insert (the coding sequence of GFP or other fluorescent protein) and sequences homologous to genomic sequences on the left and right sides of the desired site of insertion (Fig. 3; Paix, Folkmann, Goldman, et al., 2017; Paix, Folkmann, & Seydoux, 2017). Use the genomic sequence (not the cDNA sequence) when designing the donor template, as the cutting site can be close to an exon/intron junction. In addition, we recommend the following.

- The insert should be <1 kb in length, as editing frequency decreases significantly with increasing insert size (Paix, Folkmann, Goldman, et al., 2017). Most fluorescent proteins are small enough to fit this size restriction (e.g., GFP without start and stop codons is 714 bp; Supporting Information, File S1).
- The insertion site should be as close as possible to the site of Cas9-induced cleavage (Paix, Folkmann, Goldman, et al., 2017; Paquet et al., 2016). The sequence between the Cas9 cleavage site and the insertion site is homologous to the genomic region targeted (internal homology) and should be recoded with silent mutations to prevent premature recombination that would result in low insertion efficiency of the fluorescent reporter gene (incomplete editing; Fig. 3; Paix, Folkmann, Goldman, et al., 2017).
- Make sure that the donor template cannot be cut by Cas9. Mutations in the PAM sequence or in the sequence immediately upstream of the PAM in the crRNA spacer sequence are generally sufficient to prevent cleavage by Cas9 (Fig. 3; Hsu et al., 2013). Note that spyCas9 also weakly cleaves sequences with NAG or NGA PAMs, so avoid recoding the PAM to NAG or NGA (Kleinstiver et al., 2015).
- We recommend testing the donor template in silico by translating the predicted cDNA fusion in order to rule out any unwanted codon changes or frameshifts due to errors in the design of the donor template (use cloning software or online resources such as https://web.expasy.org/translate/; Gasteiger, 2003).

#### Materials

~50–100 ng of plasmid containing desired fluorescent protein sequence at ~100 ng/μl in H_2_O (Fig. 7 and Supporting Information File S1), from a standard miniprep of 1.5 ml bacterial culture (100 ng/μl in H_2_O)

Phusion Taq polymerase master mix with buffer HF and DMSO aliquots (NEB, cat. no. M0531L; store at −20°C)

Milli-Q-filtered sterile H_2_O (store at room temperature) 6× Orange G loading dye (e.g., Bioworld, cat. no. 10570024-1) Eight-PCR-tube strips with thin walls (VWR, cat. no. 93001-118) MinElute PCR Purification Kit, containing MinElute columns and PE and PB buffers (Qiagen, cat. no. 28006; add 200-proof anhydrous ethanol, e.g., Fisher Scientific, cat. no. AC615095000, to PB buffer as indicated on the buffer bottle, and store buffers at room temperature and columns at 4°C as recommended by the manufacturer)

50× TAE buffer (e.g., Thermo Fisher Scientific, cat. no. B49) Standard 1.5% agarose minigel prepared with TAE buffer and agarose (e.g., Thermo Fisher Scientific, cat. no. 16500500)

Eight-PCR-tube strips with thin walls (e.g., VWR, cat. no. 93001-118) Bench microcentrifuge (e.g., Fisher Scientific Accuspin Micro17) PCR thermocycler with temperature-gradient functionality (e.g., Bio-Rad C1000 thermal cycler)

Agarose minigel electrophoresis system (e.g., Fisher Scientific 14-955-170) NanoDrop spectrophotometer (e.g., Thermo Fisher Scientific ND-1000)

1. Design the PCR primers: Design forward and reverse primers containing ~19 nt homologous to the 5′ and 3′ ends of the desired fluorescent protein coding sequence. Add the homology arms (35-40 nt of sequence flanking the edit and cleavage site) to the 5′ end of each primers and, if necessary, for one primer, add the sequence including silent mutations between the edit and the cleavage site (Fig. 3). Purchase or synthesize the primers (e.g., 25 nmol from IDT, desalted), reconstitute each primer at 100 μM with Milli-Q-purified water, and store at −20°C.
2. Prepare PCR master mix: In a 1.5-ml microcentrifuge tube, mix 0.8 μl template plasmid, 2 μl each of 100 μM stocks of the forward and the reverse primers, 200 μl 2× Phusion Master mix, and 195.2 μl H_2_O. Vortex, and then split the mix into a PCR strip with eight tubes (50 μl per tubes).
3. Run PCR as follows:

**Table:** 

Initial step:	2 min	98°C	(denaturation)
30 cycles:	30 s	98°C	(amplification)
	30 s	61.5°C	
	45 s	72°C	
Final step:	10 min	72°C	(elongation)
Hold at 4°C.
4. Analyze PCR products: Add 10 μl 6× Orange G loading dye to the 50-μl PCR reaction and run 8 μl on a 1.5% agarose minigel.
  We found that most primers work well at an annealing temperature of 61.5°C. However, if the PCR reactions have a low yield or produce nonspecific gel bands, run a gradient PCR from 60°C to 72°C to determine the optimal annealing temperature. We also found that adding 3% DMSO (1.5 μl per 50-μl PCR reaction) can improve the specificity/yield of PCR reactions.
5. Purify PCR products: Pool the eight PCR reactions together in a 15-ml Falcon tube, add 2 ml buffer PB, and vortex. Place 750 μl in a MinElute column and spin 1 min at 16,200 × *g* (13,000 rpm), room temperature, on a bench centrifuge. Discard the flowthrough and repeat twice using the same column. Wash the column with 750 μl PE buffer (containing ethanol), spin, and discard flowthrough. Re-spin the column empty. Carefully remove the column from the collection tube and place in a 1.5-ml microcentrifuge tube. Add 10 μl of H_2_O in the middle of the column membrane (do not touch it). Wait 1–5 min and spin again. Discard the column and keep the tube containing the purified PCR donor.
  It is not necessary to digest away the template plasmid as long as the plasmid does not contain sequence that could drive expression of the fluorescent protein and lead to false positives.
  We do not recommend using agarose gel purification, as this procedure results in impure and low-yield DNA preparations.
6. Determine the concentration using 1 μl purified PCR on a NanoDrop spectrophotometer. The expected concentration is 0.8–1.3 μg/μl.

## ALTERNATE PROTOCOL 1

### gBLOCK DONOR DESIGN

As an alternative to PCR fragments (Basic Protocol 2), commercial synthesized gene fragments can be used as donors (e.g., gBlocks from IDT). gBlocks can be purchased commercially, but typically are provided in a limited quantity (1 μg for 751–1000 bp gBlocks, sufficient for one editing experiment).

#### Additional Materials (see Basic Protocol 2)

Gene fragment (e.g., IDT gBlock)

1. Design the gBlock using the same design considerations as for a PCR donor (Basic Protocol 2; Fig. 3). The sequence complexity of the homology arms may prevent efficient gBlock synthesis. If so, extend the length of the homology arms while maintaining the total size of the gBlock at <1 kb (to obtain a yield of 1 μg).
2. Order the gBlock (e.g., IDT): 1 μg, no 5′ phosphate group. Upon receiving the gBlock, do not reconstitute; simply store as is at room temperature.

## SUPPORT PROTOCOL 1

### Cas9 PROTEIN PURIFICATION

Relatively large amounts of spyCas9 protein are required for each editing experiment (80 μg/experiment for nucleofection and 8 μg/experiment for lipofection). Cas9 can be purchased from commercial suppliers, but it is also straightforward to purify in the laboratory. The procedure requires only few days and can yield enough Cas9 to perform >50 genome editing experiments using nucleofection (Basic Protocol 3).

#### Materials

Rosetta 2 competent *Escherichia coli* cells for protein expression (Novagen, cat. no. 71397)

nm2973 plasmid encoding spyCas9::SV40::6×His construct (Addgene, accession no. 67881; Fu, Hansen, Artiles, Nonet, & Fire, 2014)

LB + ampicillin plates (e.g., IPM Scientific, cat. no. 11006–002; store at 4°C) LB + 50 μg/ml carbenicililn medium (see recipe)

1 M isopropyl β-D-1-thiogalactopyranoside (IPTG; e.g., GoldBio, cat. no. 12481C5) in Milli-Q-purified H_2_O (filter and store at −20°C)

Buffer A (see recipe)

Protease inhibitor cocktail (e.g., Sigma-Aldrich, cat. no. 11836170001; use one tablet per 10 ml of solution)

Lysozyme (e.g., Sigma-Aldrich, cat. no. L6876; prepare 10 mg/ml stock in Milli-Q-purified H_2_O and store at −20°C)

Ni-NTA agarose (e.g., Qiagen, cat. no. 30410, 25 ml)

Buffer B (see recipe)

Buffer C (see recipe)

Q Sepharose (e.g., Sigma-Aldrich, cat. no. Q1126) 1 M KCl (e.g., Sigma-Aldrich, cat. no. P9541) Buffer D (see recipe)

10% polyacrylamide gels (e.g., Thermo Fisher Scientific, cat. no. NW04122BOX), optional

26/60 Sephacryl S-200 column (GE Healthcare, cat. no. 17–1195-01), optional

Bacterial incubator shakers (e.g., New Brunswick Innova 4230 and Innova 44)

Disposable OD cuvette (e.g., Fisher Scientific, cat. no. 14–955-127)

Spectrometer (e.g., Biochrom WPA CO8000)

Ultracentrifuge (e.g., Thermo-Fisher Scientific Sorval Lynx 6000), rotor (e.g., Thermo-Fisher Scientific Fiberlite F9–6X1000LEX), and compatible centrifuge bottles

Sonicator (e.g., Branson Ultrasonics Digital Sonifier 250)

Tabletop centrifuge (e.g., Eppendorf 5810R) and rotor (e.g., Eppendorf A-4–62-MTP)

0.45-μm filter cups (e.g., Foxx Life Sciences, cat. no. 1162-RLS)

5-ml chromatography columns

Fast protein liquid chromatography (FPLC) apparatus (e.g., GE Healthcare Akta Pure 25 M)

NanoDrop spectrophotometer (e.g., Thermo Fisher Scientific ND-1000)

Polyacrylamide gel apparatus: e.g., Mini Gel Tank (Thermo-Fisher Scientific, cat.

no. A25977)

Dialysis cell: e.g., Slide-A-Lyzer, 20,000 MWCO (Thermo-Fisher Scientific, cat. no. 66012)

100K centrifugal filter (Amicon, cat. no. UFC910024)

0.5-ml freezing tubes, sterile

Liquid nitrogen

Ultrafreezer, −80°C

#### Culture Cas9-producing E. coli

1. Transform 30 μl Rosetta 2 competent *E. coli* cells with nm2973 plasmid using standard procedures for chemically competent bacteria. Plate on LB + ampicillin plates and grow overnight at 37°C.
2. Pick a few colonies from the plates and use to inoculate 25 ml LB + 50 μg/ml carbenicillin medium. Grow overnight at 37°C.
3. Transfer 5 ml of the overnight culture to 1 L LB + 50 μg/ml carbenicillin and grow at 37°C. Measure OD using a spectrometer until OD_600_ = 0.5–0.7.
4. Shift culture to 18°C for 15–25 min, and then add 1 M IPTG stock to 0.2 mM. Grow culture overnight at 18°C.
5. Pellet culture for 10 min at 5422 × *g* (5000 rpm), 4°C, in a tabletop centrifuge and measure the wet weight of the pellet. Resuspend the pellet at ~6 ml/g cells with buffer A + protease inhibitor cocktail.
6. Add lysozyme to final 1 mg/ml concentration. Perform lysis and following steps on ice or at 4°C.
7. Using a sonicator, sonicate six times for 45 s each (30% power, 1 s pulse and 2 s pause), cooling on ice for 1 min between cycles.
8. Spin lysate 30 min at 29,097 × *g* (13,000 rpm), 4°C, in an ultracentrifuge and transfer supernatant to a fresh tube.
9. Filter supernatant through 0.45-μm filter cup.

#### Purify Cas9 by fast protein liquid chromatography

**Figure 8:**
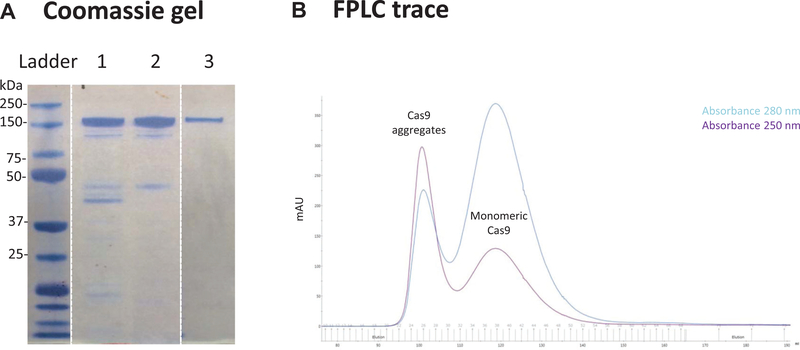
Cas9 protein purification. (**A**) Coomassie-stained SDS-PAGE gel of purified Cas9. Recombinant Cas9 is affinity purified using Ni-NTA agarose (lane 1). Pooled eluent is flowed over Q Sepharose to remove contaminating DNA bound to Cas9 (lane 2). To remove Cas9 aggregates, the eluent is further fractionated via size-exclusion chromatography S200 (lane 3). Samples are resolved by SDS-PAGE and visualized by Coomassie staining. (**B**) Size-exclusion chromatogram of Cas9. Eluted fractions from the S200 chromatography were collected and the protein absorbance was measured at 250 nm (purple) and 280 nm (blue). mAU, milli absorbance units. Fractions representing monomeric Cas9 were pooled and concentrated.

10. Prepare and equilibrate a 5-ml Ni-NTA agarose column with buffer A (at least 5 column volumes).
11. Batch bind-clarified lysate with Ni-NTA agarose 45 min at 4°C.
12. Wash Ni-NTA agarose column with at least 20 column volumes of buffer B.
13. Elute protein with buffer C. Determine which fractions contain Cas9 protein using a NanoDrop or by running a small amount on SDS gel, and pool these fractions together.
14. To remove contaminating DNA from the prep: Prepare and equilibrate a 5-ml Q Sepharose column with 25 ml 1 M KCl to charge the column. Equilibrate column with buffer C (25 ml). Flow eluent (from step 13) over prepared Q Sepharose column. Collect flowthrough and dialyze into 1 L buffer D at 4°C overnight.
15. *Optional*: To remove Cas9 aggregates from the sample, the pooled fractions (from step 14) can be further fractionated via size-exclusion chromatography:
  - Equilibrate a 26/60 Sephacryl S-200 column with 1.5 column volumes buffer D.
  - Concentrate the pooled fractions from step 14 to 5-ml volume using a 100K centrifugal filter, run the 5-ml concentrated sample over the S200 column, and collect the monomeric 280 nm absorbance peak (from the FPLC spectrometer) using the fraction collector (Fig. 8).
16. Concentrate Cas9 (from step 14 or 15) to ~10 mg/ml using a 100K centrifugal filter. Distribute in 10 μl aliquots into sterile 0.5-ml tubes. Flash freeze in liquid nitrogen. Store aliquots at −80°C.

A typical prep will yield 50–70 single-use aliquots (10-μl aliquot, 10 μg/μl Cas9; Fig. 8).

## BASIC PROTOCOL 3

### GENE TAGGING USING NUCLEOFECTION

Nucleofection is an efficient method for delivery of programmed Cas9 and donor templates into HEK293T, DLD1, or U2OS cells (Table 1). This method relies on a solution that permeabilizes the cells along with an electroporator device (Amaxa Nucleofector) used to introduce programmed Cas9 and donor template in the permeabilized cells. Lipofection is an alternative delivery technique that does not require specialized equipment, but is less efficient (Alternate Protocol 2).

#### Materials

Culture of the cells of interest, grown to 70%−80% confluence (Support Protocol 2) crRNA and tracrRNA (Basic Protocol 1)

Cas9 protein (from Support Protocol 1 for recombinant Cas9 preparation, or purchased from commercial sources: e.g., IDT, cat. no. 1081058)

Donor DNA: PCR donor DNA (Basic Protocol 2) or gBlock donor DNA (Alternate Protocol 1)

Filtered sterile glycerol/KCl buffer (see recipe)

Filtered sterile HEPES/MgCl_2_/KCl buffer (see recipe)

Milli-Q-filtered sterile H_2_O (store at 4°C)

Amaxa Cell Line Nucleofector Kit V (Lonza, cat. no. VCA-1003; add the 500 μl of the provided nucleofection supplement solution to the 2.25 ml of nucleofection reagent and store at 4°C; can be used for up to 3 months after adding the supplement solution)

Penicillin/streptomycin (Pen/Strep; e.g., Thermo-Fisher Scientific, cat. no.; store at −20°C; prepare working aliquot in 15-ml Falcon tubes and store at −20°C, then transfer 0.5 ml in 50 ml culture medium into a 50-ml Falcon tube and store at 4°C)

75-cm^2^ (T75) ventilated culture flasks (e.g., USA Scientific, cat. no. CC7682–4175) 6-well plate (e.g., Corning, cat. no. 3516 or 3506)

22 × 22–mm coverslips (e.g., Fisher Scientific, cat. no. 22–050-218), sterilized under UV or under flame with ethanol

Narrow-end pipets (EMS, cat. no. 70967–14S)

Electroporation cuvettes (provided with Amaxa nucleofector kit) Amaxa Nucleofector 2b device (Lonza, AAB-1001)

Tabletop centrifuge (e.g., Eppendorf, 5810R) and rotors (e.g., Eppendorf, A-4–62-MTP and FA-45–30-11)

Additional reagents and equipment for cell culture (Support Protocol 2)

#### Grow cells for nucleofection (3–4 days before nucleofection)

1. Prepare two T75 culture flasks containing 12 ml of appropriate cell culture medium (enough for 6–8 experiments with HEK293T and DLD1 cells or 3–4 experiments for U2OS cells).
2. From a culture grown to 70%−80% confluence, harvest the cells with trypsin (Support Protocol 2). Seed the cells into the two T75 flasks (500,000 HEK293T cells, 650,000 DLD1 cells, or 800,000 U2OS cells per flask). Place the cells back in the cell incubator.
  Cells can be transformed by nucleofection 3–4 days after seeding (at 50%−70% confluence).

#### Prepare the dsDNA donor mix for nucleofection (on the day of nucleofection)

3a. *If using a PCR donor*: Dilute 6–8 μg of the purified PCR product in 10 μl H_2_O final. Keep on ice or at room temperature. For a PCR donor containing GFP and ~35-bp homology arms, this concentration will be sufficient to obtain a final concentration of 0.2–0.3 μM in the final 50 μl Cas9 RNP/donor mix. To adjust for larger or small donor templates, use the following formula: 1 μg of X bp dsDNA = 1624/X pmol.
  A final donor DNA concentration of 0.3 μM is recommended for highest yield (Fig. 4).
3b. *If using a gBlock donor (1 μg)*: Spin briefly in a bench microcentrifuge at room temperature. Keep at room temperature.
  Spin down cells for 3 min at 350 × g (1426 rpm) with A-4–62-MTP rotor or at 350 × g (1815 rpm) with FA-45–30-11 rotor.

#### Prepare the Cas9 RNP mix for nucleofection (on the day of nucleofection)

4. Prepare the Cas9 RNP mix immediately before nucleofection. Place the crRNA, tracrRNA, and Cas9 protein on ice. The homemade Cas9 aliquots (Support Protocol 1) are intended to be single use but can be flash frozen again at least one more time without loss of activity. Take the number of Cas9 aliquots needed, briefly spin them in a bench microcentrifuge at room temperature, pool Cas9 into one tube, and store on ice.
5. Equilibrate the glycerol/KCl buffer, HEPES/MgCl_2_/KCl buffer, and Milli-Q-purified H_2_O at room temperature.
6. In a 1.5-ml microcentrifuge tube, mix reagents in the following order (If several experiments are planned, make all the necessary Cas9 RNP mixes):
  12 μl glycerol/KCl buffer
  5 μl HEPES/MgCl_2_/KCl buffer
  5 μl 65 μM crRNA
  5 μl crRNA tracrRNA
  8 μl 10 μg/μl Cas9 protein 5 μl Milli-Q-purified H_2_O.
  *Optional*: Incubate the mix at 37°C before placing on ice to encourage Cas9 RNP complex formation.
7. Store on ice.
  Optional: the Cas9 RNP mix can be kept up to 45 min at room temperature.

#### Harvest cells for nucleofection (the day of nucleofection)

8. Resuspend the cells at 50%−70% confluence from two T75 flasks: use trypsin as described in Support Protocol 2 (except using 3× more PBS, trypsin, and culture medium for T75 flasks than T25 flasks).
9. Resuspend the pellet in 1 ml PBS and transfer into a 1.5-ml microcentrifuge tube.
10. Take 10 μl of cells, dilute in 90 μl of PBS, and remove 10 μl for cell counting.
11. Count the cells (the cell concentration is 10 times that from the count because of the dilution in step 10). Spin down the cells for 3 min at 350 × *g* (1815 rpm with FA-45–30-11 rotor), room temperature (1 ml in PBS in a 1.5-ml microcentrifuge tube).
12. Remove the supernatant (from step 3) and resuspend the cells in PBS to a concentration of 1 × 106 cells/ml (from 0.8 × 106 to 1.2 × 106 is also acceptable).
13. Aliquot 80 μl of resuspended cells (from step 12) each into 1.5-ml microcentrifuge tubes (one tube per editing experiment + one additional tube for a no-transformation negative control), and spin down the cells for 3 min at 350 × *g* (1815 rpm with FA-45–30-11 rotor), room temperature. Keep the tubes at room temperature without removing the supernatant.
14. Prepare 6-well plates to receive the transformed cells: one plate per experiment + one for no-transformation negative control. Fill each of four wells per plate with 2 ml culture medium. The wells will be used for cell counting by cytometer (well no. 1) and for microscopy analysis (well no. 2; add coverslip to the well). The two extra wells can be used to passage the transformed cells and/or to derive single-cell clones. Also prepare 1.5-ml microcentrifuge tubes containing 600 μl culture medium (to recover the cells after nucleofection; one tube per experiment). Keep the plates and tubes in the cell incubator.

#### Nucleofection procedure

15. Equilibrate the nucleofector solution at room temperature.
16. Move the 6-well plates and the 1.5-ml microcentrifuge tubes with culture medium (prepared to receive the transformed cells) from the cell incubator into the cell culture hood.
17. Remove the supernatant of the cell pellet from step 13 (80 μl), and add 80 μl of nucleofection solution (solution V with supplement solution added). Mix by pipetting.
18a. *If using a PCR donor*: Take 40 μl Cas9 RNP mix (from step 7) and add it to 10 μl of the PCR donor mix, and mix by pipetting.
18b. *If using a gBlock donor*: Take 40 μl Cas9 RNP mix (from step 7) and add it to the tube containing the dried gBlock donor, spin briefly in a bench microcentrifuge at room temperature, wait 1 min, and mix by pipetting.
19. Take 40 μl of the Cas9 RNP/donor mix and add it to the 80 μl of cells in the nucleofection solution.
20. Using a narrow-end pipet, gently mix the nucleofection mix containing the cells + nucleofection solution and Cas9 RNP/donor mix, and transfer it in the bottom of an Amaxa 2b electroporation cuvette.
  Do not make bubbles and avoid transferring the mix outside the space between the two electrodes.
21. Put the lid on the cuvette and tap it on a solid surface in order to remove any bubbles, and place the cuvette in the Amaxa 2b machine and run the appropriate electroporation program (A023 for HEK293T and DLD1 cells, X001 for U2OS cells).
22. Remove the cuvette from the Amaxa 2b machine, remove the lid, and immediately add the 600 μl of culture medium from the 1.5-ml microcentrifuge tube (using a micropipet with 1000-μl tip) and mix by pipetting. Transfer the culture medium with cells back into the 1.5-ml microcentrifuge tube (using a fresh narrow-end pipet). Next, transfer 150 μl cells in culture medium into each of four wells of the 6-well plate (using a micropipet with 200-μl tip) and replace the plates in the cell incubator.
23. Repeat with other Cas9 RNP/donor mixes as needed (from step 16). For the negative control, resuspend the 80 μl of cells and place 10 μl in the wells of a 6-well plate.
24. The next day, replace the culture medium of the transformed cells with fresh medium containing Pen/Strep. If necessary, the cells can also be split into several wells using trypsin treatment.

## Note for two-genes or two-colors tagging using nucleofection

**Figure 9:**
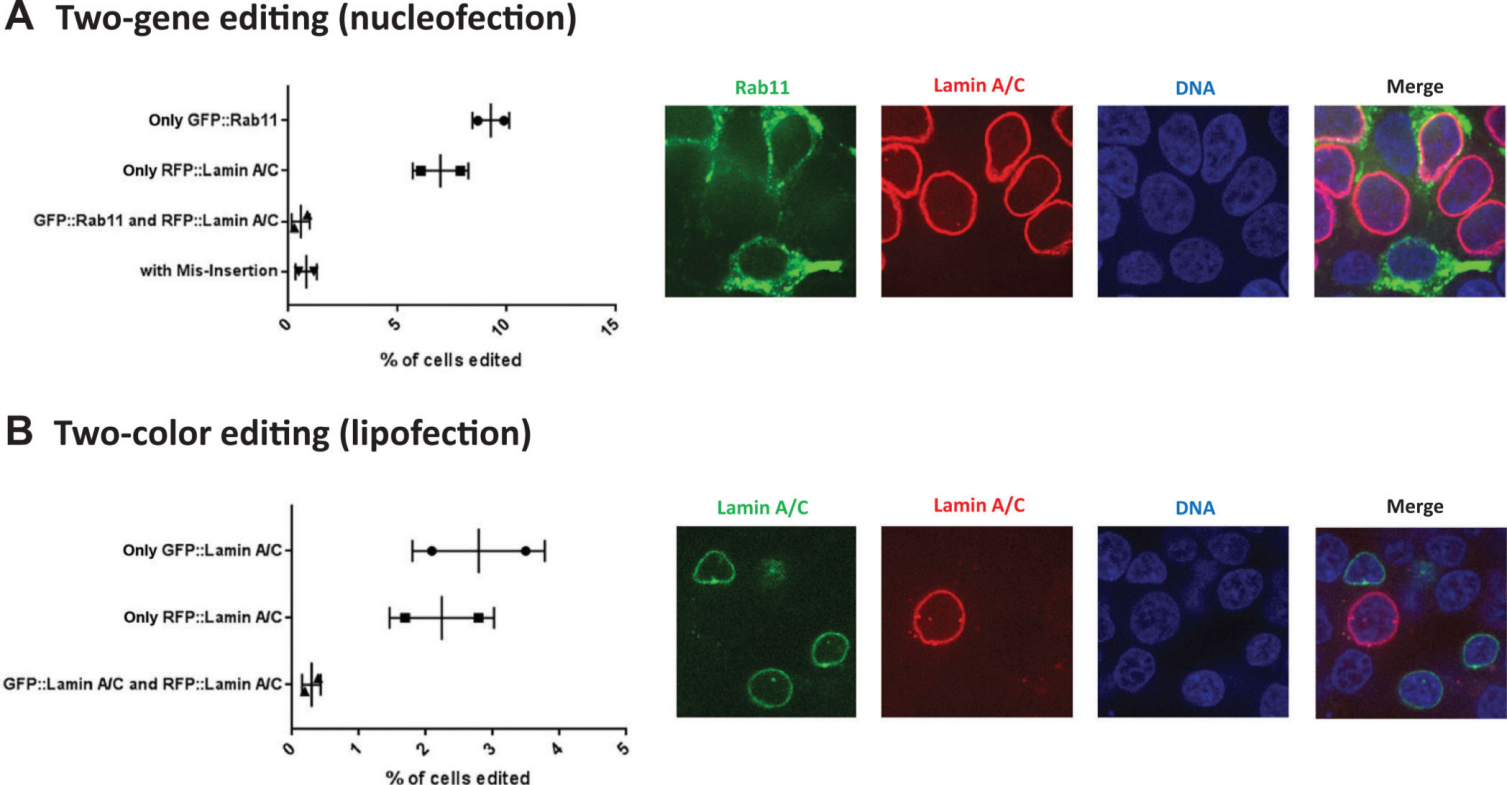
Efficiency of multiplex tagging using nucleofection and lipofection. Graphs show the percentage of cells edited (average with standard deviation) as indicated and images show examples of edited cells. (**A**) Experiment targeting two loci (*Rab11* and *Lamin A/C*, HEK293T cells) with GFP and RFP, respectively. Tagged cells exhibit GFP signal in endosomes (green) and/or nuclear membrane RFP signal (red). Mis-insertion indicates rare events where the wrong tag was inserted at either locus. Because each editing event is independent, co-edits are rare but do occur. In the example shown, a few co-targeted cells are visible (red and green in same cell). (**B**) Experiment targeting the same locus (*Lamin A/C*, HEK293T cells) with GFP and RFP. This approach can be used to generate cells where the targeted locus is tagged with GFP or RFP in one experiment. Occasionally, cells where both alleles are targeted by the different tags can be recovered, but this is a rare event (see graph). No such cells are visible in the example shown.

25a. *To tag two genes with two fluorescent proteins* (Fig. 9): For the Cas9 RNP, mix the two different crRNAs targeting the two desired genes (2.5 μl of each crRNA at 65 μM). For the donor mix, use two repair donors encoding the two different fluorescent proteins at a 1:1 molar ratio. Be sure that the two donors do not contain any similar sequences and encode fluorescent proteins with distinct excitation and emission spectra (such as eGFP and mCherry).
25b. *To tag one locus with two fluorescent proteins* (Fig. 9): Prepare the Cas9 RNP with a single crRNA. For the dsDNA donor mix, use two repair donors coding for the two different fluorescent proteins at a 1:1 molarity ratio.

## ALTERNATE PROTOCOL 2

### GENE TAGGING USING LIPOFECTION

Lipofection relies on liposome particles that passively fuse with cells to deliver the Cas9 RNP/Donor mix. This method does not require specialized equipment but is less efficient than nucleofection (Basic Protocol 3). We recommend lipofection for cell types that transform efficiently, such as HEK293T (Table 2).

*IMPORTANT NOTE*: To tag two genes with two fluorescent proteins using lipofection, or one gene with two fluorescent proteins (Fig. 9), see Basic Protocol 3. When tagging two genes, use 0.25 μl of each crRNA at 65 μM.

#### Materials

Culture of the cells of interest, grown to 70%−80% confluence (Support Protocol 2)

Cas9 protein (from Support Protocol 1 for recombinant Cas9 preparation, or purchased from commercial sources, e.g., IDT, cat. no. 1081058)

crRNA and tracrRNA (Basic Protocol 1)

Donor DNA: PCR donor DNA (Basic Protocol 2) or gBlock donor DNA (Alternate Protocol 1)

Filtered sterile glycerol/KCl buffer (see recipe)

Filtered sterile HEPES/MgCl_2_/KCl buffer (see recipe)

Milli-Q-filtered sterile H_2_O (stored at 4°C)

Penicillin/streptomycin (Pen/Strep; e.g., Thermo-Fisher Scientific, cat. no.

15140–122; store at −20°C; prepare working aliquot in 15-ml Falcon tubes and store at −20°C, then transfer 0.5 ml in 50 ml culture medium into a 50-ml Falcon tube and store at 4°C)

LipoJet In Vitro Transfection kit version II with 5× Lipofection buffer and LipoJet reagent (SignaGen Laboratories, cat. no. SL100468; store at 4°C)

6-well plate (Corning, e.g., cat. no. 3516 or 3506)

Additional reagents and equipment for cell culture (Support Protocol 2)

#### Grow cells for lipofection (2–3 days before lipofection)

1. From a culture grown to 70%−80% confluence, harvest the cells with trypsin (Support Protocol 2).
2. Add 2 ml culture medium per well into a 6-well plate. Each well corresponds to one editing experiment. Add coverslips to the wells if needed for microscopy analysis.
3. Seed the cells in each well (e.g., 65,000/well for HEK293T cells). Place the cells back in the cell incubator. Cells can be transformed by lipofection 2–3 days after seeding (50%−70% confluence).

#### Prepare dsDNA donor mix for lipofection (on the day of lipofection)

4a. *If using a PCR donor*: Use 0.8–1.0 μg/μl purified PCR product (diluted in H2O, 1 μl per each lipofection experiment). Spin briefly in a bench microcentrifuge at room temperature and keep at room temperature. A final concentration of 0.3–0.4 μM is desired in the 5 μl of Cas9 RNP/donor mix (Fig. 4). For donor DNAs significantly larger or smaller than GFP, consult Basic Protocol 3 to calculate concentrations.
4b. *If using a gBlock donor*: Add 1 μl H_2_O on the wall of the gBlock tube and spin briefly in a bench microcentrifuge at room temperature. Keep at room temperature.

#### Prepare the Cas9 RNP mix for lipofection (on the day of lipofection)

5. Prepare the Cas9 RNP mix immediately before starting the lipofection experiment.

Store crRNA, tracrRNA, and Cas9 protein on ice.

6. Equilibrate the glycerol/KCl buffer, HEPES/MgCl_2_/KCl buffer, and Milli-Q-purified H_2_O at room temperature. Mix the Cas9 RNP reagents in a 1.5-ml microcentrifuge tube in the following order (If several experiments are planned, make all the necessary Cas9 RNP mixes):
  1.2 μl glycerol/KCl buffer
  0.5 μl HEPES/MgCl_2_/KCl buffer
  0.5 μl crRNA (65 μM)
  0.5 μl tracrRNA (65 μM)
  0.8 μl Cas9 protein (10 μg/μl)
  0.5 μl H_2_O.
  *Optional*: Incubate the mix 15 min at 37°C to encourage Cas9 RNP complex formation.

7. Store at room temperature.

#### Lipofection procedure

8. Equilibrate the 5× lipofection buffer, LipoJet reagent, and Milli-Q-filtered H_2_O at room temperature.
9. Prepare the 1× lipofection buffer by diluting 40 μl 5× stock buffer (shake to dissolve any precipitate) in 160 μl of H_2_O in a 1.5-ml microcentrifuge tube. Make a master mix if several experiments are planned.
10. Add 4 μl Cas9 RNP mix to 1 μl of dsDNA donor mix (PCR or gBlock, from step 4a or 4b) and mix by pipetting.
11. Add 4 μl of the Cas9 RNP/donor mix (from step 10) to 200 μl of 1× lipofection buffer, and immediately add 5 μl of LipoJet reagent. Mix by pipetting and incubate 10 min at room temperature.
12. Add the Cas9 RNP/donor/LipoJet mix (from step 11) to the cells in one well of a 6-well plate. Mix by shaking the plate gently and place back in the incubator. If several experiments are planned, steps 10–12 can be performed in parallel.
13. The next day, replace the culture medium of the transformed cells with fresh medium containing Pen/Strep. If necessary, the cells can also be split into several wells using trypsin.

#### SUPPORT PROTOCOL 2

## MAINTENANCE AND PREPARATION OF TISSUE CULTURE CELLS FOR TRANSFORMATION

We recommend the following guidelines for tissue culture handling:

- *Sterile conditions*: It is important to work under sterile conditions when manipulating cell lines and culture reagents. We use antibiotic (Pen/Strep) in the cell culture medium after cell transformation because the Cas9, crRNA, tracrRNA, and donor DNA may not be sterile.
- *Cell adherence*: HEK293T, DLD1 and U2OS are adherent cells. They can be detached using trypsin treatment. An incubation of 1–2 min followed by pipetting will be enough for HEK293T cells, whereas up to 7–10 min will be necessary for DLD1 and U2OS cells. HEK293T can also be partially detached by routine pipetting, and thus with these cells it is necessary to exercise caution when changing medium.
- *Cell passaging*: We limit the number of cell passages to minimize any genetic drift (Liu et al., 2019). After 30 passages, thaw a frozen cell stock vial to obtain fresh cells (Basic Protocol 4).

### Materials

DMEM medium (e.g., Thermo Fisher Scientific, cat. no. 11995–065; store at 4°C)

FBS, one-shot 50-ml vial (e.g., Thermo Fisher Scientific, cat. no. A31604–02; store keep at −20°C)

Culture medium: add a one-shot 50-ml FBS vial to DMEM medium and store at 4°C

Cultured HEK293T (ATCC cell line provider, cat. no. CRL-11268, Research Resource IDentifier RRID:CVCL_1926), DLD1 (ATCC, cat. no. CCL-221, RRID:CVCL_0248), or U2OS cells (ATCC, cat. no. HTB-96, RRID:CVCL_0042)

1× PBS (e.g., Thermo Fisher Scientific, cat. no. 20012–027; store at room temperature when unopened and at 4°C when opened)

Trypsin-EDTA (e.g., Thermo Fisher Scientific, cat. no. 25200–072; store at −20°C, and make several working aliquots of 35 ml each in 50-ml Falcon tubes and store those at −20°C)

0.4% Trypan Blue dye (e.g., Bio-Rad, cat. no. 1450021)

Cell incubator (e.g., Thermo Fisher Scientific Heracell VIOS 160i), 5% CO_2_, 37°C

Cell culture hood (e.g., The Baker Company SterilGard SG503A-HE)

Ventilated 25-cm^2^ (T25) flask (e.g., USA Scientific, cat. no. CC7682–4825)

Cell counting chambers (e.g., Bio-Rad, cat. no. 1450011)

Tabletop centrifuge (e.g., Eppendorf 5810R) and rotors (e.g., Eppendorf A-4–62-MTP and FA-45–30-11)

Cell counter (e.g., Bio-Rad TC20)

1. Equilibrate the cell culture reagents at room temperature.
2. When the cells reach 70%−80% confluence (every 4–5 days) in a T25 flask, prepare one T25 flask containing 4 ml culture medium.
3. Wash the cells with 2 ml 1× PBS.
4. Add 2 ml trypsin-EDTA and wait until the cells become nonadherent.
5. Resuspend the cells and transfer them in a 15-ml Falcon tube containing 2 ml culture medium.
6. Spin down the cells for 3 min at 350 × *g* (1426 rpm with A-4–62-MTP rotor), room temperature.
7. Remove the supernatant and resuspend the cells in 4 ml culture medium with FBS.
8. Transfer 1 ml of the resuspended cells in a 1.5-ml microcentrifuge tube. From the microcentrifuge tube, take 10 μl of resuspended cells and transfer it in a fresh microcentrifuge tube for counting cells.
9. Determine the cell concentration: add 10 μl 0.4% Trypan Blue dye to the 10 μl cell suspension, and count cells using a cell counter.
10. Seed the cells at the desired concentration into the T25 flask (150,000 cells for HEK293T, 200,000 cells for DLD1, 230,000 cells for U2OS). Place the cells back in the cell incubator.

## BASIC PROTOCOL 4

### ANALYSIS OF NUCLEOFECTED/LIPOFECTED CELLS

Three days after transformation (Basic Protocol 3 and Alternate Protocol 2), cells can be analyzed by cytometry or microscopy to determine editing efficiency and the expression pattern of the fluorescent reporter fusion. Positive cells can be further sorted and cloned for subsequent analyses, and frozen for long term storage.

#### Materials

Transformed cells (Basic Protocol 3 or Alternate Protocol 2)

Formaldehyde solution in PBS: prepare by mixing 10 ml 16% (w/v) formaldehyde (e.g., Thermo Fisher Scientific, cat. no. 28908) with 30 ml of 1× PBS (store at 4°C)

Triton X-100 (e.g., Sigma-Aldrich, cat. no. T8787)

PBS/0.25% Triton: prepare by making 10% (v/v) Triton X-100 (e.g.,

Sigma-Aldrich, cat. no. T8787) in H_2_O and mixing 1 ml with 39 ml PBS (store at room temperature)

Antifade mounting medium with DAPI (e.g., Vectashield, Vector Laboratories, cat. no. H-1200; store at 4°C)

DMSO (e.g., Sigma-Aldrich, cat. no. D2438; store at room temperature)

Tabletop centrifuge (e.g., Eppendorf 5810R) and rotors (e.g., Eppendorf A-4–62-MTP and FA-45–30-11)

1.5-ml tube without cap (e.g., Fisher Scientific, cat. no. 02–681-339)

Cytometer (e.g., Millipore Guava EasyCyte 6/2 L)

Microscope slides (e.g., VWR, cat. no. 48312–004)

Clear fingernail polish

Test tubes with cell strainer snap cap (e.g., Corning, cat. no. 352235)

96-well plates (e.g., Corning, cat. no. 3595)

Cryogenic vials (e.g., Corning, cat. no. 430488)

Freezing container (e.g., Thermo Fisher Scientific, cat. no. 5100–0001)

Fluorescent microscope (with light source compatible with the fluorescent reporter used)

Fluorescence-activated cell sorter FACS) at a local flow cytometry facility Liquid nitrogen

Ultrafreezer, −80°C

Additional reagents and equipment for cell culture (Support Protocol 2)

#### Calculate percentage of positive cells using cytometry

1. Equilibrate the cell culture reagents (medium with FBS, PBS, and trypsin) at room temperature.
2. At 60–72 hr post cell transformation, the cells can be analyzed for fluorescent signal. Wash one well containing the transformed cells with 1 ml PBS.
3. Add 1 ml trypsin and resuspend the cells.
4. Nontransformed cells are used as control.
5. Transfer the resuspended cells in a 1.5-ml microcentrifuge tube containing 500 μl culture medium.
6. Spin down the cells for 3 min at 350 × *g* (1815 rpm with FA-45–30-11 rotor), room temperature.
7. Remove supernatant and resuspend the cells in 1 ml PBS.
8. Briefly vortex resuspended cells in PBS and immediately transfer 400 μl into a 1.5-ml tube without a cap.
9. Run appropriate cleaning and calibration procedures through the cytometer. Set the cytometer to detect the fluorescent reporter of interest.
10. Run nonfluorescent cells (nontransformed cells) to determine the fluorescence background and to check the cytometer parameters.
11. Measure the fluorescent reporter expression in the transformed cells. We recommend analyzing 10,000 cells per sample, vortex the cells before every run. If the cells concentration exceeds 1500 cells/μl, dilute in PBS.
12. Analyze the results: Determine the cell population to analyze on the negative control, and count fluorescent cells. We recommend setting a background threshold where <0.1% of the cells in the negative control are counted as GFP positive (false positive rate; Figs. 4–6).

#### Analyze the expression pattern of the fluorescent reporter using microscopy

12. Equilibrate the PBS/formaldehyde solution at room temperature.
13. At 60–72 hr post cell transformation, the cells can be analyzed for fluorescent signal. From the culture well containing a coverslip with cells grown on it, transfer the coverslip to a fresh 6-well plate containing PBS.
  Nontransformed cells are used as control.
14. Remove the PBS and incubate 1 hr with PBS/formaldehyde solution.
15. Wash with PBS.
16. Wash with PBS/0.25% Triton for 5 min, and then again with PBS.
17. Mount coverslips on microscope slides using antifade mounting medium (containing DAPI). If necessary, remove excess PBS from the coverslip with paper tissue before mounting.
18. Seal with clear fingernail polish.
19. Analyze cells for fluorescent reporter expression by fluorescent microscopy. The total number of cells can be determined using the DAPI nuclear dye contained in the mounting medium.

#### Cell sort using FACS

20. Equilibrate cell culture reagents to room temperature.
21. Harvest edited cell population using trypsin (Support Protocol 2) and resuspend in PBS + Pen/Strep at 1 × 106 cells/ml.
  Non-transformed cells are used as control.
22. Prepare two 96-well plates for cell cloning (two plates are generally enough to recover >75 viable clones) with 100 μl of culture medium + Pen/Strep in each well. If the sorted cells are pooled together instead of cloned in plate wells, prepare a 1.5-ml microcentrifuge tube containing 500 ml culture medium with Pen/Strep.
23. Filter the cells with cell strainers and keep the tubes on ice.
24. Sort the fluorescence-positive cells according to the FACS instructions.
25. Incubate the plates in a cell incubator. After 72 hr, add 125 μl culture medium with Pen/Strep to each well. If the sorted cells have been pooled in a single tube, transfer the contents of the tube into a well of a 6-well plate containing 1.5 ml culture medium with Pen/Strep, and change culture medium 3 days later (again using culture medium with Pen/Strep).
26. Genotype single-cell clones using PCR primers. Use primers from outside the donor sequence to avoid false positives from off-target insertions (Won & Dawid, 2017).
  Pooled sorted cells can be analyzed by microscopy or subcloned if necessary.

#### Cell freezing

27. Grow the cells in a T25 flask to 70%−80% confluence.
28. Equilibrate cell culture reagents to room temperature.
29. Harvest cells using trypsin (Support Protocol 2) and resuspend in culture medium at 10 × 106 cells/ml.
30. Prepare 2 ml freezing medium containing 75% culture medium + 25% DMSO. Transfer 400-μl aliquots of freezing medium into five cryogenic vials.
31. Add 100 μl cell suspension to each cryogenic vial and mix by pipetting.
32. Put the tubes in a slow-freezing container and store overnight at −80°C.
33. Transfer the tubes into a liquid nitrogen tank.

#### Cell thawing protocol

34. Add 10 ml culture medium to a 15-ml Falcon tube and warm to 37°C.
35. Transfer the cryogenic vials from the liquid nitrogen storage and warm them in your hand. As soon as the culture medium is liquid, transfer the cells to the Falcon tube containing warm culture medium. Spin down the cells for 3 min at 350 × *g* (1426 rpm with A-4–62-MTP rotor), room temperature, and resuspend in 2 ml culture medium.
36. Transfer the 2 ml cells into a well of a 6-well plate. The following day, change the culture medium (Pen/Strep can be included if needed).

## REAGENTS AND SOLUTIONS

Use gloves and clean labware throughout to avoid contamination.

### Buffer A (20 mM Tris, pH 8.0, 250 mM KCl, 20 mM imidazole, 10% glycerol, 1 mM TCEP)

2.42 g Tris·HCl (e.g., Thermo Fisher Scientific, cat. no. 17926) 800 ml Milli-Q-purified H_2_O

Adjust pH to 8.0 with NaOH

18.68 g KCl (e.g., Sigma-Aldrich, cat. no. P9541)

1.36 g imidazole (e.g., Sigma-Aldrich, cat. no. I2399)

100 ml glycerol (e.g., Sigma-Aldrich, cat. no. G6279) Bring volume to 1 L with Milli-Q-purified H_2_O

Pass through a 0.22-μM filter and store at 4°C

Add 0.5 M tris(2-carboxyethyl)phosphine (TCEP; e.g., Thermo Fisher Scientific, cat. no. 77720) to a final concentration of 1 mM just before using buffer A (TCEP is expensive, so add it only to the volume of buffer A needed).

### Buffer B (20 mM Tris, pH 8.0, 800 mM KCl, 20 mM imidazole, 10% glycerol, 1 mM TCEP)

2.42 g Tris·HCl (e.g., Thermo Fisher Scientific, cat. no. 17926) 800 ml of Milli-Q-purified H_2_O

Adjust pH to 8.0 with NaOH

59.64 g KCl (e.g., Sigma-Aldrich, cat. no. P9541)

1.36 g imidazole (e.g., Sigma-Aldrich, cat. no. I2399) 100 ml glycerol (e.g., Sigma-Aldrich, cat. no. G6279) Bring volume to 1 L with Milli-Q-purified H_2_O

Pass through a 0.22-μM filter and store at 4°C

Add TCEP to a final concentration of 1 mM before using buffer B (TCEP is expensive, so add it only to the volume of buffer B needed).

### Buffer C (20 mM HEPES, pH 8.0, 500 mM KCl, 250 mM imidazole, 10% glycerol)

4.79 g HEPES (e.g., Sigma-Aldrich, cat. no. H7006)

800 ml of Milli-Q-purified H_2_O

Adjust pH to 8.0 with NaOH

37.27 g KCl (e.g., Sigma-Aldrich, cat. no. P9541)

17 g imidazole (e.g., Sigma-Aldrich, cat. no. I2399)

100 ml glycerol (e.g., Sigma-Aldrich, cat. no. G6279)

Bring volume to 1 L with Milli-Q-purified H_2_O

Pass through a 0.22-μM filter and store at 4°C.

### Buffer D (20 mM HEPES, pH 7.5, 500 mM KCl, 20% glycerol)

4.79 g HEPES (e.g., Sigma-Aldrich, cat. no. H7006)

800 ml Milli-Q-purified H_2_O

Adjust pH to 7.5 with NaOH

37.27 g KCl (e.g., Sigma-Aldrich, cat. no. P9541)

200 ml glycerol (e.g., Sigma-Aldrich, cat. no. G6279)

Bring volume to 1 L with Milli-Q-purified H_2_O

Pass through a 0.22-μM filter and store at 4°C.

### Glycerol/KCl buffer (30% glycerol, 150 mM KCl)

300 ml glycerol (e.g., Sigma-Aldrich, cat. no. G6279)

11.18 g KCl (e.g., Sigma-Aldrich, cat. no. P9541)

Bring volume to 1 L with Milli-Q-purified H_2_O. Pass through a 0.22-μM filter and store at 4°C.

### HEPES/MgCl_2_/KCl buffer (20 mM HEPES, pH 7.4, 10 mM MgCl_2_, 150 mM KCl)

4.79 g HEPES (e.g., Sigma-Aldrich, cat. no. H7006)

0.9 g MgCl_2_ (e.g., Sigma-Aldrich, cat. no. M8266)

11.08 g KCl (e.g., Sigma-Aldrich, cat. no. P9541)

Bring volume to 1 L with Milli-Q-purified H_2_O

Adjust pH to 7.4 with NaOH

Pass through a 0.22-μM filter and store at 4°C.

### LB + 50 μg/ml carbenicillin medium

Prepare LB mix (e.g., Genesee Scientific, cat. no. 11–118) at 25 g/L in Milli-Q-purified H2O, autoclave, and store at room temperature. Prepare 1000× stock of carbenicillin (e.g., Sigma-Aldrich, cat. no. C1389) at 50 mg/μl in Milli-Q-purified H2O, filter, and store at −20°C. Add carbenicillin to LB just before inoculating bacteria.

## COMMENTARY

## Background Information

The genome engineering method presented here is supported by a prior study from our group that examined how linear donor DNAs engage with the cell’s machinery for HDR to repair Cas9-induced double-strand breaks (Paix, Folkmann, Goldman, et al., 2017). Our findings suggest that donor DNAs are not integrated at Cas9-induced DNA breaks, but are used as templates for repair synthesis.

The repair process involves several steps (Fig. 2), including:

1. Resection of the cleavage site (by 5′ exonucleases to expose single-stranded 3′ ends on either side of the break),
2. Pairing of the resected ends with the donor DNA,
3. Repair synthesis that extends the 3′ resected ends using the donor as a template,
4. Template switching which allows the newly synthesized strand to pair with the resected end on the other side of the lesion,
5. Fill-in synthesis and ligation.

Two key features of the repair process inform the design of the donor DNA. First, annealing of the donor DNA to the resected ends at the locus can be achieved with relatively short homology arms on the donor DNA. Homology arms that are 35 nucleotides (nt) long are sufficient for efficient pairing and initiation of repair synthesis (Paix, Folkmann, Goldman, et al., 2017). Second, frequent template switching during repair synthesis requires the donor DNA to be designed in such a way as to minimize any homology between the edit and the cleavage site, as template switching in this region will prevent incorporation of the edit (Paix, Folkmann, Goldman, et al., 2017). For this reason, it is important to minimize the distance between the edit and the Cas9 cleavage site (<10 bp is ideal). Recoding of the sequence between the edit and the Cas9 cleavage site also can be used to help reduce premature template switching (Basic Protocol 2 and Fig. 3). Even with recoding, however, we have found that distances greater than 10 bp do not yield high editing efficiencies (Paix, Folkmann, Goldman, et al., 2017).

It is important to keep in mind that most fluorescent reporter insertions obtained by this protocol will target only one allele. The other allele (or alleles depending on the cell type) may be unaffected or may contain small insertions and/or deletions created by a Cas9 cleavage event that was repaired by nonhomologous end joining (NHEJ; Paix, Folkmann, Goldman, et al., 2017). For this reason, it is important when deriving clonal populations to sequence both alleles to ensure there are no unwanted mutations.

In regard to reagent costs, the main cost is associated with the custom crRNA (~$100 U.S.). Donor DNA costs range between ~$25 for PCR donors and ~$100 for gBlock donors. The cost of Cas9 protein is negligible when Cas9 in produced in house, and the cost associated with the tracrRNA is a few dollars per experiment. The cost of other disposable reagents amounts to $50–200 per experiment depending of the delivery method used and the types of cell analyses performed. The suppliers referenced in this protocol were the ones used to develop this genome engineering method; alternative suppliers may be used as preferred.

## Critical Parameters and Troubleshooting

No edits or very low editing efficiency may be due to the following.

### Low crRNA efficiency.

We recommend a crRNA with 50% GC content and a 3′ G in the spacer sequence that will cleave within a 10-bp window of the desired fluorescent reporter insertion site (Doench et al., 2014; Gagnon et al., 2014; Paix, Folkmann, Goldman, et al., 2017; Paquet et al., 2016).

### Low concentration of donor.

A concentration >0.2 μM (~0.6 μg/μl of a PCR donor containing GFP) is recommended for maximal efficiency (Fig. 4; Paix, Folkmann, Goldman, et al., 2017).

### Precipitation of Cas9 in the Cas9 RNP/donor mix.

We recommend adding each reagent in the order specified in Basic Protocol 3 and Alternate Protocol 2 to avoid precipitation of Cas9.

### Overgrown cell culture.

Avoid using cells that have reached confluence. We recommend using cells at no more than 75% confluence for transformation.

### Gene is not expressed or expressed at a very low level.

Because the identification of the edits depends on fluorescent reporter expression, make sure that the gene of interest is expressed in the cell type that you will use for your editing experiment. The Protein Atlas database provides useful expression information for a number of commonly used tissue culture cells (https://www.proteinatlas.org/; Uhlen et al., 2015). Keep in mind that fluorescent reporter fusions with low, diffuse expression may be difficult to detect above background in a cytometer. We recommend direct observation of the transformed cells by microscopy to avoid missing low-expression fusions.

### Fusion is not stable.

In some cases, the fusion may interfere with protein stability or protein dynamics (Nagasaki et al., 2017). A flexible linker sequence (Chen, Zaro, & Shen, 2013) can be added to the insert in order to improve folding of the tagged protein. A different insertion site can also be tested.

## Understanding Results

When targeting a site within 10 bp of the Cas9 cleavage site, depending of the locus targeted, the delivery method, and the cell type used, researchers can expect editing efficiencies ranging from 0.5% to 20% (Tables 1 and 2 and Supporting Information, Table S1). This range is suitable for localization studies in nonclonal populations, and for isolation of edited clonal populations from sorted single cells.

We recommend including a positive control when using the protocol for the first time.

### Positive control for PCR donor.

Use the *Lamin A/C* crRNA (crRNA AP1629; Supporting Information, Table S3), eGFP forward and reverse primers (primer AP1618 and AP1619; Supporting Information, Table S2) and GFP plasmid pAP1698 (available at AddGene; Fig. 7). Perform a PCR as described in Basic Protocol 2 using an annealing temperature of 61.5°C and an elongation step of 45 sec. Purify on a MinElute column and measure the concentration with a NanoDrop (expected concentration should be >1000 ng/μl). Use 7 μg for nucleofection or 1 μg for lipofection. Expected efficiency is ~15% for nucleofection in HEK293T cells (Fig. 4 and Table 1).

### Positive control for gBlock donor.

Order the *HIST2H2BE* crRNA (crRNA AP3311; Supporting Information, Table S3), the eGFP gBlock (gBlock AP3320; Supporting Information, Table S2). Use 1 μg of the provided gBlock for nucleofection. Expected efficiency is 1.5% for nucleofection in HEK293T cells (Fig. 5 and Table 1).

## Time Considerations

### Gathering of reagents.

5–7 days in advance of the nucleofection or lipofection, users should design and order the crRNA, and the PCR primers or gBlock required for the donor. The other reagents can be ordered in bulk (tracrRNA and Cas9) or synthesized in the lab (Cas9) and used for several experiments. Tissue culture cells are passaged 2–4 days before transformation.

### Cell transformation and analyses.

Only 0.5–2 hr of hands-on work is needed is for transformation and plating of the edited cells. Cytometer and microscopy analyses are performed 2–3 days later.

## Supplementary Material

File S1

Table S1

Table S2

Table S3

## Figures and Tables

**Table 1:** Summary of Gene Tagging Experiments using Nucleofection

Gene	Cell type	Repair template	crRNA (polarity)	Homology arms (bp)	[C] template (μM)	Tagging position (relative to Cas9 cut)	% efficiency (GFP^+^)

*Lamin A/C*	HEK293T	PCR	1629 (S)	33/33	0.33	Nt (cut)	15.7 (±3.0, *n* = 6)
*Lamin A/C*	HEK293T	PCR	1629 (S)	0/0	0.33	Nt (cut)	1.2 (±1.0, *n* = 3)
*Rab11*	HEK293T	PCR	1648 (AS)	33/33	0.33	Nt (cut)	15.6 (±6.9, *n* = 2)
*Rab11*	HEK293T	PCR	1648 (AS)	0/0	0.33	Nt (cut)	0.7 (±0.1, *n* = 2)
*Nup35*	HEK293T	PCR	1656 (S)	33/33	0.33	Ct (−13)	5.5 (±0.6, *n* = 2)
*Nup35*	HEK293T	PCR	1656 (S)	0/0	0.33	Ct (cut)	1.0 (*n* = 1)
*CBX1*	HEK293T	PCR	1646 (S)	35/35	0.33	Nt (+3)	4.5 (±0.7, *n* = 2)
*CBX1*	HEK293T	PCR	1646 (S)	0/0	0.33	Nt (cut)	0.9 (*n* = 1)
*Sec61B*	HEK293T	PCR	1647 (S)	33/35	0.21	Nt (+1)	2.6 (±0.8, *n* = 2)
*Sec61B*	HEK293T	PCR	1647 (S)	0/0	0.21	Nt (cut)	0.6 (*n* = 1)
*SMC3*	HEK293T	PCR	1553 (AS)	37/38	0.33	Ct (+5)	15.5 (±6.4, *n* = 3)
*Lamin A/C*	HEK293T	gBlock	1629 (S)	0/0	0.05	Nt (cut)	0.3 (*n* = 1)
*Lamin A/C*	HEK293T	gBlock	1629 (S)	33/33	0.05	Nt (cut)	5.3 (±1.8, *n* = 2)
*Lamin A/C*	HEK293T	gBlock	1629 (S)	101/103	0.04	Nt (cut)	5.1 (*n* = 1)
*VIM*	HEK293T	gBlock	3313 (AS)	39/39	0.05	Nt (+5)	5.9 (*n* = 1)
*MAPRE1*	HEK293T	gBlock	3312 (AS)	38/39	0.05	Nt (+12)	1.7 (*n* = 1)
*HIST2H2BE*	HEK293T	gBlock	3311 (AS)	39/39	0.05	Ct (+1)	1.3 (*n* = 1)
*G3BP1*	HEK293T	gBlock	3310 (S)	36/36	0.05	Nt (−1)	6.2 (*n* = 1)
*ACTB*	HEK293T	gBlock	3314 (S)	38/38	0.05	Nt (+5)	4.0 (*n* = 1)
*ARL6IP1*	HEK293T	gBlock	3309 (S)	39/39	0.05	Nt (−1)	3.0 (*n* = 1)
*Lamin A/C*	U2OS	PCR	1629 (S)	33/33	0.33	Nt (cut)	1.7 (±1.3, *n* = 3)
*Lamin A/C*	U2OS	PCR	1629 (S)	0/0	0.33	Nt (cut)	0.2 (±0.2, *n* = 2)
*Rab11*	U2OS	PCR	1648 (AS)	33/33	0.33	Nt (cut)	1.0 (±0.2, *n* = 3)
*Rab11*	U2OS	PCR	1648 (AS)	0/0	0.33	Nt (cut)	0.2 (±0.2, *n* = 3)
*Lamin A/C*	U2OS	gBlock	1629 (S)	29/29	0.05	Nt (cut)	0.2 (*n* = 1)
*HIST2H2BE*	U2OS	gBlock	3311 (AS)	39/39	0.05	Ct (+1)	0.2 (*n* = 1)
*VIM*	U2OS	gBlock	3313 (AS)	29/41	0.05	Nt (+5)	0.4 (*n* = 1)
*MAPRE1*	U2OS	gBlock	3312 (AS)	38/39	0.05	Nt (+12)	0.1 (*n* = 1)
*Lamin A/C*	DLD1	PCR	1629 (S)	33/33	0.33	Nt (cut)	0.3 (±0.3, *n* = 3)
*Lamin A/C*	DLD1	PCR	1629 (S)	0/0	0.33	Nt (cut)	0.1 (±0.1, *n* = 2)
*Rab11*	DLD1	PCR	1648 (AS)	33/33	0.33	Nt (cut)	0.4 (±0.2, *n* = 2)
*Rab11*	DLD1	PCR	1648 (AS)	0/0	0.33	Nt (cut)	0.0 (±0.0, *n* = 2)
*Lamin A/C*	DLD1	gBlock	1629 (S)	29/29	0.05	Nt (cut)	0.3 (*n* = 1)
*ACTB*	DLD1	gBlock	3314 (S)	38/38	0.05	Nt (+5)	0.6 (*n* = 1)
*ARL6IP1*	DLD1	gBlock	3309 (S)	39/39	0.05	Nt (−1)	0.5 (*n* = 1)
*G3BP1*	DLD1	gBlock	3310 (S)	36/36	0.05	Nt (−1)	0.1 (*n* = 1)

**Table 2:** Summary of Gene Tagging Experiments using Lipofection

Gene	Cell type	Repair template	crRNA (polarity)	Homology arms (bp)	[C] template (μM)	Tagging position (relative to Cas9 cut)	% efficiency (GFP^+^)

*Lamin A/C*	HEK293T	PCR	1629 (S)	33/33	0.41	Nt (cut)	6.8 (±1.4, *n* = 4)
*Lamin A/C*	HEK293T	PCR	1629 (S)	0/0	0.41	Nt (cut)	1.2 (±0.1, *n* = 2)
*Rab11*	HEK293T	PCR	1648 (AS)	33/33	0.41	Nt (cut)	7.7 (±1.3, *n* = 2)
*Rab11*	HEK293T	PCR	1648 (AS)	0/0	0.41	Nt (cut)	0.6 (±0.4, *n* = 2)
*Nup35*	HEK293T	PCR	1656 (S)	33/33	0.39	Ct (−13)	1.3 (*n* = 1)
*CBX1*	HEK293T	PCR	1646 (S)	35/35	0.39	Nt (+3)	3.2 (*n* = 1)
*ACTB*	HEK293T	PCR	3302 (S)	36/35	0.41	Ct (−1)	7.4 (±0.4, *n* = 2)
*TUBG1*	HEK293T	PCR	3300 (S)	33/33	0.36	Ct (−7)	0.7 (±0.1, *n* = 2)
*SMC3*	HEK293T	PCR	1553 (AS)	37/38	0.41	Ct (+5)	4.8 (±2.8, *n* = 2)
*Lamin A/C*	HEK293T	gBlock	1629 (S)	33/33	0.41	Nt (cut)	2.4 (±3.2, *n* = 3)
*Lamin A/C*	HEK293T	gBlock	1629 (S)	101/103	0.35	Nt (cut)	3.9 (±3.7, *n* = 3)
*Lamin A/C*	HEK293T	gBlock	1629 (S)	0/0	0.22	Nt (cut)	0.8 (*n* = 1)
*Nup35*	HEK293T	gBlock	1656 (S)	33/33	0.40	Ct (−13)	0.7 (*n* = 1)
*Nup35*	HEK293T	gBlock	1656 (S)	64/68	0.37	Ct (−13)	0.1 (*n* = 1)
*CBX1*	HEK293T	gBlock	1646 (S)	35/35	0.41	Nt (+3)	0.4 (*n* = 1)
*CBX1*	HEK293T	gBlock	1646 (S)	65/64	0.38	Nt (+3)	1.1 (*n* = 1)
*Sec61B*	HEK293T	gBlock	1647 (S)	40/40	0.40	Nt (+4)	3.2 (*n* = 1)
*Lamin A/C*	U2OS	PCR	1629 (S)	33/33	0.41	Nt (cut)	0.0 (±0.0, *n* = 3)
*Lamin A/C*	U2OS	PCR	1629 (S)	0/0	0.41	Nt (cut)	0.0 (±0.0, *n* = 2)
*Rab11*	U2OS	PCR	1648 (AS)	33/33	0.41	Nt (cut)	0.1 (±0.1, *n* = 3)
*Rab11*	U2OS	PCR	1648 (AS)	0/0	0.41	Nt (cut)	0.0 (±0.0, *n* = 2)
*Lamin A/C*	DLD1	PCR	1629 (S)	33/33	0.41	Nt (cut)	0.0 (±0.0, *n* = 2)
*Lamin A/C*	DLD1	PCR	1629 (S)	0/0	0.41	Nt (cut)	0.0 (±0.0, *n* = 2)
*Rab11*	DLD1	PCR	1648 (AS)	33/33	0.41	Nt (cut)	0.1 (±0.1, *n* = 2)
*Rab11*	DLD1	PCR	1648 (AS)	0/0	0.41	Nt (cut)	0.0 (±0.0, *n* = 2)
