# Supplementary material for: Rapid Tagging of Human Proteins with Fluorescent Reporters by Genome Engineering using Double-Stranded DNA Donors
Source: Curr Protoc Mol Biol. Author manuscript; Available in PMC 2020 Dec 1. (PMC6935516; doi:10.1002/cpmb.102)
Supplement: File S1 [file NIHMS1059829-supplement-File_S1.docx]

**File S1**

**Sequences coding for fluorescent proteins and other tags optimized for mammalian expression**

All inserts were cloned from gBlocks containing 2*15bp homology sequences with *BamH1* digested pUC19 (in yellow). Plasmids will be deposed at Addgene.

>pAP1638: 4*(GFP11)

cggtacccggggatcAGAGACCACATGGTTTTGCATGAGTATGTGAACGCGGCGGGTATAACTGGTGGGTCGGGCGGACGAGACCATATGGTGCTTCACGAATACGTAAACGCAGCTGGCATTACTGGCGGATCAGGTGGCAGGGATCACATGGTACTCCATGAGTACGTGAACGCTGCTGGAATCACAGGCGGTAGCGGCGGTCGGGACCATATGGTCCTGCACGAATATGTCAATGCTGCCGGTATCACCGGAAGTTCCGGCGGCgatcctctagagtcg

>pAP1680: tagRFP

cggtacccggggatcGTCTCTAAAGGGGAAGAACTCATCAAGGAGAATATGCACATGAAGCTCTATATGGAAGGTACAGTAAACAATCACCACTTCAAATGCACTTCTGAAGGTGAAGGAAAGCCCTACGAGGGTACACAGACGATGAGAATTAAAGTCGTTGAGGGAGGCCCGCTGCCCTTTGCTTTTGACATCCTTGCAACCTCATTTATGTATGGCAGCCGAACATTCATCAATCATACACAGGGGATTCCAGATTTTTTCAAACAGTCCTTTCCGGAGGGCTTTACATGGGAGCGAGTAACGACCTATGAAGATGGGGGAGTTTTGACCGCCACGCAAGATACGTCACTCCAAGACGGCTGCCTGATTTACAATGTAAAAATAAGAGGCGTTAATTTTCCGTCTAATGGCCCGGTAATGCAGAAAAAGACCCTGGGTTGGGAGGCCAACACGGAAATGCTTTATCCGGCAGACGGTGGCTTGGAGGGGCGCTCTGACATGGCACTGAAGTTGGTCGGCGGAGGTCACCTTATTTGCAACTTCAAAACTACGTACCGCTCAAAGAAGCCAGCCAAAAACCTTAAAATGCCCGGGGTTTATTATGTAGACCACCGCCTCGAACGGATCAAGGAAGCTGATAAAGAAACGTATGTAGAGCAACATGAAGTCGCAGTGGCAAGGTACTGTGATCTCCCCAGTAAGCTGGGGCACAAACTGAATgatcctctagagtcg

>pAP1698: eGFP

cggtacccggggatcGTGAGCAAGGGCGAGGAGCTGTTCACCGGGGTGGTGCCCATCCTGGTCGAGCTGGACGGCGACGTAAACGGCCACAAGTTCAGCGTGTCCGGCGAGGGCGAGGGCGATGCCACCTACGGCAAGCTGACCCTGAAGTTCATCTGCACCACCGGCAAGCTGCCCGTGCCCTGGCCCACCCTCGTGACCACCCTGACCTACGGCGTGCAGTGCTTCAGCCGCTACCCCGACCACATGAAGCAGCACGACTTCTTCAAGTCCGCCATGCCCGAAGGCTACGTCCAGGAGCGCACCATCTTCTTCAAGGACGACGGCAACTACAAGACCCGCGCCGAGGTGAAGTTCGAGGGCGACACCCTGGTGAACCGCATCGAGCTGAAGGGCATCGACTTCAAGGAGGACGGCAACATCCTGGGGCACAAGCTGGAGTACAACTACAACAGCCACAACGTCTATATCATGGCCGACAAGCAGAAGAACGGCATCAAGGTGAACTTCAAGATCCGCCACAACATCGAGGACGGCAGCGTGCAGCTCGCCGACCACTACCAGCAGAACACCCCCATCGGCGACGGCCCCGTGCTGCTGCCCGACAACCACTACCTGAGCACCCAGTCCGCCCTGAGCAAAGACCCCAACGAGAAGCGCGATCACATGGTCCTGCTGGAGTTCGTGACCGCCGCCGGGATCACTCTCGGCATGGACGAGCTGTACAAGgatcctctagagtcg

>pAP1836: mCherry

cggtacccggggatcGTGAGCAAGGGCGAGGAGGATAACATGGCCATCATCAAGGAGTTCATGCGCTTCAAGGTGCACATGGAGGGCTCCGTGAACGGCCACGAGTTCGAGATCGAGGGCGAGGGCGAGGGCCGCCCCTACGAGGGCACCCAGACCGCCAAGCTGAAGGTGACCAAGGGTGGCCCCCTGCCCTTCGCCTGGGACATCCTGTCCCCTCAGTTCATGTACGGCTCCAAGGCCTACGTGAAGCACCCCGCCGACATCCCCGACTACTTGAAGCTGTCCTTCCCCGAGGGCTTCAAGTGGGAGCGCGTGATGAACTTCGAGGACGGCGGCGTGGTGACCGTGACCCAGGACTCCTCCCTGCAGGACGGCGAGTTCATCTACAAGGTGAAGCTGCGCGGCACCAACTTCCCCTCCGACGGCCCCGTAATGCAGAAGAAGACCATGGGCTGGGAGGCCTCCTCCGAGCGGATGTACCCCGAGGACGGCGCCCTGAAGGGCGAGATCAAGCAGAGGCTGAAGCTGAAGGACGGCGGCCACTACGACGCTGAGGTCAAGACCACCTACAAGGCCAAGAAGCCCGTGCAGCTGCCCGGCGCCTACAACGTCAACATCAAGTTGGACATCACCTCCCACAACGAGGACTACACCATCGTGGAACAGTACGAACGCGCCGAGGGCCGCCACTCTACCGGCGGCATGGACGAGCTGTACAAGgatcctctagagtcg

>pAP1837: mCherry (alternative sequence)

cggtacccggggatcGTCTCTAAAGGAGAAGAAGATAATATGGCTATCATTAAAGAGTTTATGCGATTCAAAGTGCACATGGAAGGAAGTGTCAACGGCCATGAATTCGAAATCGAAGGGGAAGGAGAAGGTCGCCCCTATGAAGGCACTCAGACGGCAAAACTTAAGGTTACAAAGGGAGGTCCGCTTCCCTTCGCGTGGGATATACTTTCTCCTCAGTTCATGTATGGGTCCAAAGCCTACGTCAAGCATCCAGCAGACATTCCGGATTATCTGAAGCTGTCATTTCCCGAGGGTTTTAAATGGGAAAGGGTGATGAATTTCGAGGACGGGGGAGTTGTGACCGTTACACAGGATTCTTCCCTTCAGGACGGCGAGTTCATATATAAAGTCAAGCTCCGAGGGACAAACTTCCCAAGTGATGGACCTGTCATGCAAAAGAAGACCATGGGCTGGGAAGCTTCTTCAGAACGAATGTATCCAGAAGACGGAGCGCTGAAAGGTGAGATAAAGCAGAGGTTGAAACTGAAGGATGGGGGGCATTACGATGCAGAAGTGAAAACCACATATAAAGCAAAGAAACCCGTACAACTGCCAGGAGCATATAATGTCAATATCAAACTTGATATTACCAGCCACAATGAAGACTACACTATAGTTGAGCAATACGAAAGGGCAGAGGGACGGCATTCCACAGGCGGAATGGATGAACTCTATAAAgatcctctagagtcg

>pAP1894: TEV::eGFP::Linker::Partial-mNeonGreen::3xFlag::tagRFP

cggtacccggggatcGAGAACCTCTACTTCCAAGGAGTGAGCAAGGGCGAGGAGCTGTTCACTGGTGTCGTTCCGATTCTCGTGGAATTGGATGGAGATGTAAATGGTCATAAATTTTCTGTTTCCGGGGAGGGGGAGGGCGACGCTACTTATGGGAAGCTGACATTGAAATTTATATGCACAACTGGTAAGCTCCCTGTGCCGTGGCCCACCCTTGTAACGACCCTCACATACGGCGTCCAGTGCTTTAGCCGCTACCCCGATCACATGAAACAGCATGACTTCTTTAAGTCCGCGATGCCTGAGGGATACGTCCAAGAAAGGACGATCTTCTTTAAGGATGACGGTAACTATAAAACCAGGGCCGAGGTTAAGTTTGAGGGTGATACACTTGTAAACCGAATAGAACTGAAGGGTATAGATTTTAAAGAGGATGGAAATATCCTTGGCCATAAACTTGAATATAACTATAATTCCCACAATGTATATATTATGGCTGACAAGCAAAAGAATGGTATAAAAGTTAATTTCAAGATCCGACACAACATAGAAGATGGCAGCGTACAATTGGCGGACCATTACCAGCAAAACACACCCATCGGCGACGGACCAGTGCTTTTGCCCGACAACCATTATTTGTCTACACAGAGCGCGCTTAGCAAGGACCCTAATGAGAAACGAGATCATATGGTCCTTCTTGAATTTGTTACGGCCGCTGGGATCACTCTCGGCATGGACGAGCTGTACAAGTGCCCCGGTGACCGATGGTCCTCTACTGGTGGCGGGCGCTCTCGCACAAGTGACAACATGGCATCTTTGCCAGCGACGCACGAATTGCACATATTTGGCTCAATTAACGGAGTGGACTTTGATATGGTGGGTCAGGGCACTGGCAATCCTAACGACGGGTACGAGGAACTTAACCTCAAATCCACGAAGGGCGATCTCCAGTTTTCCCCTTGGATTCTGGTCCCCCATATTGGGTACGGGTTCCACCAGTACCTCCCATACCCGGACGGTATGAGCCCGTTCCAAGCGGCTATGGTGGATGGTAGCGGGTACCAGGTCCATAGAACCATGCAGTTCGAAGATGGTGCCAGTCTTACGGTGAACTACAGGTACACGTATGAAGGCTCACACATTAAGGGGGAGGCGCAGGTCAAAGGTACGGGTTTCCCGGCGGATGGACCCGTGATGACTAACTCCCTGACCGCAGCAGATTGGTGCCGCTCAAAAAAAACCTATCCGAATGATAAAACGATTATAAGTACGTTCAAGTGGTCTTATACAACTGGGAACGGTAAGAGGTACAGGTCAACCGCTAGAACAACATACACCTTTGCGAAGCCAATGGCAGCTAACTACCTGAAGAACCAACCAATGTACGTCTTTAGAAAGACTGAACTGAAGCATAGCAAAACTGAGTTGAATTTTAAAGAATGGCAAAAGGCTTTTACGGACGTTATGGGTGACTACAAAGATCACGACGGCGATTACAAGGACCATGACATAGATTACAAGGACGATGATGACAAGGTATCTAAAGGGGAAGAACTCATCAAGGAGAATATGCACATGAAGCTCTATATGGAAGGTACAGTAAACAATCACCACTTCAAATGCACTTCTGAAGGTGAAGGAAAGCCCTACGAGGGTACACAGACGATGAGAATTAAAGTCGTTGAGGGAGGCCCGCTGCCCTTTGCTTTTGACATCCTTGCAACCTCATTTATGTATGGCAGCCGAACATTCATCAATCATACACAGGGGATTCCAGATTTTTTCAAACAGTCCTTTCCGGAGGGCTTTACATGGGAGCGAGTAACGACCTATGAAGATGGGGGAGTTTTGACCGCCACGCAAGATACGTCACTCCAAGACGGCTGCCTGATTTACAATGTAAAAATAAGAGGCGTTAATTTTCCGTCTAATGGCCCGGTAATGCAGAAAAAGACCCTGGGTTGGGAGGCCAACACGGAAATGCTTTATCCGGCAGACGGTGGCTTGGAGGGGCGCTCTGACATGGCACTGAAGTTGGTCGGCGGAGGTCACCTTATTTGCAACTTCAAAACTACGTACCGCTCAAAGAAGCCAGCCAAAAACCTTAAAATGCCCGGGGTTTATTATGTAGACCACCGCCTCGAACGGATCAAGGAAGCTGATAAAGAAACGTATGTAGAGCAACATGAAGTCGCAGTGGCAAGGTACTGTGATCTCCCCAGTAAGCTGGGGCACAAACTGAATgatcctctagagtcg

>pAP2041: eGFP::3xFlag::mCherry

cggtacccggggatcGTGAGCAAGGGCGAGGAGCTGTTCACCGGGGTGGTGCCCATCCTGGTCGAGCTGGACGGCGACGTAAACGGCCACAAGTTCAGCGTGTCCGGCGAGGGCGAGGGCGATGCCACCTACGGCAAGCTGACCCTGAAGTTCATCTGCACCACCGGCAAGCTGCCCGTGCCCTGGCCCACCCTCGTGACCACCCTGACCTACGGCGTGCAGTGCTTCAGCCGCTACCCCGACCACATGAAGCAGCACGACTTCTTCAAGTCCGCCATGCCCGAAGGCTACGTCCAGGAGCGCACCATCTTCTTCAAGGACGACGGCAACTACAAGACCCGCGCCGAGGTGAAGTTCGAGGGCGACACCCTGGTGAACCGCATCGAGCTGAAGGGCATCGACTTCAAGGAGGACGGCAACATCCTGGGGCACAAGCTGGAGTACAACTACAACAGCCACAACGTCTATATCATGGCCGACAAGCAGAAGAACGGCATCAAGGTGAACTTCAAGATCCGCCACAACATCGAGGACGGCAGCGTGCAGCTCGCCGACCACTACCAGCAGAACACCCCCATCGGCGACGGCCCCGTGCTGCTGCCCGACAACCACTACCTGAGCACCCAGTCCGCCCTGAGCAAAGACCCCAACGAGAAGCGCGATCACATGGTCCTGCTGGAGTTCGTGACCGCCGCCGGGATCACTCTCGGCATGGACGAGCTGTACAAGGACTACAAAGACCATGACGGTGATTATAAAGATCATGATATCGATTACAAGGATGACGATGACAAGGTCTCTAAAGGAGAAGAAGATAATATGGCTATCATTAAAGAGTTTATGCGATTCAAAGTGCACATGGAAGGAAGTGTCAACGGCCATGAATTCGAAATCGAAGGGGAAGGAGAAGGTCGCCCCTATGAAGGCACTCAGACGGCAAAACTTAAGGTTACAAAGGGAGGTCCGCTTCCCTTCGCGTGGGATATACTTTCTCCTCAGTTCATGTATGGGTCCAAAGCCTACGTCAAGCATCCAGCAGACATTCCGGATTATCTGAAGCTGTCATTTCCCGAGGGTTTTAAATGGGAAAGGGTGATGAATTTCGAGGACGGGGGAGTTGTGACCGTTACACAGGATTCTTCCCTTCAGGACGGCGAGTTCATATATAAAGTCAAGCTCCGAGGGACAAACTTCCCAAGTGATGGACCTGTCATGCAAAAGAAGACCATGGGCTGGGAAGCTTCTTCAGAACGAATGTATCCAGAAGACGGAGCGCTGAAAGGTGAGATAAAGCAGAGGTTGAAACTGAAGGATGGGGGGCATTACGATGCAGAAGTGAAAACCACATATAAAGCAAAGAAACCCGTACAACTGCCAGGAGCATATAATGTCAATATCAAACTTGATATTACCAGCCACAATGAAGACTACACTATAGTTGAGCAATACGAAAGGGCAGAGGGACGGCATTCCACAGGCGGAATGGATGAACTCTATAAAgatcctctagagtcg

>pAP2042: TEV::3xFlag::Ollas::Myc::V5::HAtag::Linker::eGFP

cggtacccggggatcGAGAACCTTTATTTTCAGGGAGACTATAAGGACCATGACGGGGATTATAAAGATCACGACATTGATTACAAGGACGACGACGATAAGTCCGGCTTCGCTAACGAATTGGGGCCAAGGCTCATGGGCAAGGAACAGAAATTGATCTCTGAGGAGGACCTGGGTAAACCTATCCCAAATCCCCTGTTGGGTTTGGACTCAACTTACCCCTACGACGTTCCTGACTATGCTTGCCCTGGTGACCGCTGGAGTTCAACAGGAGGCGGCAGATCAAGAACGAGTGTGAGCAAGGGCGAGGAGCTGTTCACCGGGGTGGTGCCCATCCTGGTCGAGCTGGACGGCGACGTAAACGGCCACAAGTTCAGCGTGTCCGGCGAGGGCGAGGGCGATGCCACCTACGGCAAGCTGACCCTGAAGTTCATCTGCACCACCGGCAAGCTGCCCGTGCCCTGGCCCACCCTCGTGACCACCCTGACCTACGGCGTGCAGTGCTTCAGCCGCTACCCCGACCACATGAAGCAGCACGACTTCTTCAAGTCCGCCATGCCCGAAGGCTACGTCCAGGAGCGCACCATCTTCTTCAAGGACGACGGCAACTACAAGACCCGCGCCGAGGTGAAGTTCGAGGGCGACACCCTGGTGAACCGCATCGAGCTGAAGGGCATCGACTTCAAGGAGGACGGCAACATCCTGGGGCACAAGCTGGAGTACAACTACAACAGCCACAACGTCTATATCATGGCCGACAAGCAGAAGAACGGCATCAAGGTGAACTTCAAGATCCGCCACAACATCGAGGACGGCAGCGTGCAGCTCGCCGACCACTACCAGCAGAACACCCCCATCGGCGACGGCCCCGTGCTGCTGCCCGACAACCACTACCTGAGCACCCAGTCCGCCCTGAGCAAAGACCCCAACGAGAAGCGCGATCACATGGTCCTGCTGGAGTTCGTGACCGCCGCCGGGATCACTCTCGGCATGGACGAGCTGTACAAGgatcctctagagtcg

>pAP2050: GFP11::TEV::3xFlag::Ollas::Myc::V5::HAtag::Linker

cggtacccggggatcCGTGACCACATGGTCCTTCATGAGTATGTAAATGCTGCTGGGATTACAGGTGGCGGCGAGAACCTTTATTTTCAGGGAGACTATAAGGACCATGACGGGGATTATAAAGATCACGACATTGATTACAAGGACGACGACGATAAGTCCGGCTTCGCTAACGAATTGGGGCCAAGGCTCATGGGCAAGGAACAGAAATTGATCTCTGAGGAGGACCTGGGTAAACCTATCCCAAATCCCCTGTTGGGTTTGGACTCAACTTACCCCTACGACGTTCCTGACTATGCTTGCCCTGGTGACCGCTGGAGTTCAACAAGAAGCCGCAGATCAAGAACGAGTgatcctctagagtcg

>pAP3041: GFP11::3xFlag::tagRFP

cggtacccggggatcCGTGACCACATGGTCCTTCATGAGTATGTAAATGCTGCTGGGATTACAGGTGGCGGCGACTACAAAGATCACGACGGCGATTACAAGGACCATGACATAGATTACAAGGACGATGATGACAAGGTATCTAAAGGGGAAGAACTCATCAAGGAGAATATGCACATGAAGCTCTATATGGAAGGTACAGTAAACAATCACCACTTCAAATGCACTTCTGAAGGTGAAGGAAAGCCCTACGAGGGTACACAGACGATGAGAATTAAAGTCGTTGAGGGAGGCCCGCTGCCCTTTGCTTTTGACATCCTTGCAACCTCATTTATGTATGGCAGCCGAACATTCATCAATCATACACAGGGGATTCCAGATTTTTTCAAACAGTCCTTTCCGGAGGGCTTTACATGGGAGCGAGTAACGACCTATGAAGATGGGGGAGTTTTGACCGCCACGCAAGATACGTCACTCCAAGACGGCTGCCTGATTTACAATGTAAAAATAAGAGGCGTTAATTTTCCGTCTAATGGCCCGGTAATGCAGAAAAAGACCCTGGGTTGGGAGGCCAACACGGAAATGCTTTATCCGGCAGACGGTGGCTTGGAGGGGCGCTCTGACATGGCACTGAAGTTGGTCGGCGGAGGTCACCTTATTTGCAACTTCAAAACTACGTACCGCTCAAAGAAGCCAGCCAAAAACCTTAAAATGCCCGGGGTTTATTATGTAGACCACCGCCTCGAACGGATCAAGGAAGCTGATAAAGAAACGTATGTAGAGCAACATGAAGTCGCAGTGGCAAGGTACTGTGATCTCCCCAGTAAGCTGGGGCACAAACTGAATgatcctctagagtcg
